# Eosinophils‐Induced Lumican Secretion by Synovial Fibroblasts Alleviates Cartilage Degradation via the TGF‐β Pathway Mediated by Anxa1 Binding

**DOI:** 10.1002/advs.202416030

**Published:** 2025-03-24

**Authors:** Wenqian Chen, Yuwei Zhou, Wenxiu Yuan, Yanjing Ou, Hanyu Lin, Kaixun He, Xueshen Qian, Huachen Chen, Chengchaozi Wang, Jie Lu, Weiping Chen, Dexiong Li, Jiang Chen

**Affiliations:** ^1^ Fujian Key Laboratory of Oral Diseases & Fujian Provincial Engineering Research Center of Oral Biomaterial & Stomatological Key Lab of Fujian College and University School and Hospital of Stomatology Fujian Medical University Fuzhou Fujian 350002 P. R. China; ^2^ Institute of Stomatology & Research Center of Dental and Craniofacial Implants School and Hospital of Stomatology Fujian Medical University Fuzhou Fujian 350002 P. R. China; ^3^ Department of Orthodontics School and Hospital of Stomatology Fujian Medical University Fuzhou Fujian 350002 P. R. China

**Keywords:** Anxa1, cartilage, eosinophils, Lumican, temporomandibular joint osteoarthritis, TGFβ2

## Abstract

The innate immune response is crucial in the progression of temporomandibular joint osteoarthritis (TMJOA). Yet, the roles of eosinophils in TMJOA remain unclear, underscoring the need for further investigation into their potential impact and mechanism. Addressing the clinical observation that eosinophil numbers in synovial fluid are higher in healthy individuals than in those with TMJOA, the vital regulation of this cell population in TMJOA by using an ovalbumin (OVA)‐induced hyper‐eosinophilia asthma rats is explored and a rat model of antibody‐mediated eosinophil depletion in vivo, and co‐culture system of synovial fibroblasts, chondrocytes, and eosinophils in vitro. The abnormal synovial proliferation, cartilage degradation, and subchondral bone erosion are effectively inhibited in OVA‐induced asthmatic rats appearing in the local accumulation of eosinophils in the synovium. Conversely, the reduction in synovial eosinophils exacerbated TMJOA in rats treated with TRFK. Mechanistically, the protective effect of eosinophils against TMJOA is attributed to their promotion of Lumican secretion in the synovium, where Lumican binds to Annexin A1 in chondrocytes, inhibits transforming growth factor β2 Annexin A1 and Smad2/3 phosphorylation. These results illustrate OVA/IL‐5‐induced eosinophils’ crucial role in TMJOA, identifying Lumican as a key anti‐TMJOA target. Collectively, these findings revealed the signature and mechanism in eosinophils that stimulate TMJOA resolution.

## Graphical abstract

Eosinophils play a crucial role in the progression of temporomandibular joint osteoarthritis (TMJOA). This study demonstrates that eosinophils, induced by OVA/IL‐5, promote Lumican secretion in the synovium, which binds to Annexin A1 in chondrocytes, inhibiting transforming growth factor β2 and Smad2/3 phosphorylation. This interaction alleviates synovial inflammation, cartilage degradation, and bone erosion, highlighting Lumican stimulated by eosinophils as a potential therapeutic target for TMJOA.

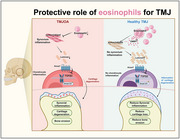

## 1 Introduction

Temporomandibular disorder (TMD) is one of the most common musculoskeletal disorders,^[^ ^1^ ^]^ with an incidence of 49.8% to 52.5% in the elderly^[^ ^2^ ^]^ and an estimated cost of $400 billion worldwide for TMD treatment.^[^ ^3^ ^]^ Temporomandibular joint osteoarthritis (TMJOA) is the most severe form of TMD, characterized by progressive cartilage degradation, subchondral bone sclerosis, synovitis, and bone changes like osteophyte formation. These symptoms impair chewing and speaking, cause chronic pain, and lead to functional limitations, significantly reducing the quality of the patient's life.^[^ ^1^, ^4^ ^]^ Traditional treatments like electric stimulation, hot compresses, intra‐articular injections, and arthroscopic surgery only provide temporary symptom relief and often come with risks such as infection, injury,^[^ ^5^ ^]^ and uncertain long‐term outcomes, sometimes even necessitating secondary surgery.^[^ ^6^ ^]^ This highlights the urgent need for more effective and targeted treatment strategies.

The activation of innate immunity is closely related to the onset and progression of osteoarthritis (OA).^[^ ^7^ ^]^ Macrophages, neutrophils, eosinophils (EOS), and other innate immune cells are present in the temporomandibular joint (TMJ) microenvironment.^[^ ^8^ ^]^ These cells recognize pathogen‐associated molecular via pattern recognition receptors,^[^ ^9^ ^]^ triggering the release of proinflammatory mediators and initiating nonspecific immune responses, which are crucial in the early inflammatory stages of OA.^[^ ^9^, ^10^ ^]^ Targeted immune‐related drug delivery using biomaterial‐based systems offers a promising approach for future TMJOA treatment.^[^ ^11^ ^]^ Previous research on TMJOA has primarily focused on macrophages, while other innate immune cells, such as eosinophils, have received insufficient attention. Eosinophils, initially thought to mediate allergic reactions and parasitic defense solely, are now recognized as critical immune‐regulatory cells.^[^ ^12^, ^13^ ^]^ In addition to bacterial killing,^[^ ^14^, ^15^ ^]^ eosinophils enhance T‐cell responses,^[^ ^16^, ^17^ ^]^ promote tissue repair,^[^ ^18^, ^19^ ^]^ and modulate inflammation by inhibiting Th17 cells via IL‐1Ra secretion and expressing regulatory molecules such as CD80 and PD‐L1.^[^ ^20^ ^]^ Besides, due to variations in their expression profiles, subpopulations, and environment, eosinophils may exert distinct functions in different settings.^[^ ^20^, ^21^ ^]^ Eosinophils, though few, are resident in the synovium. Genomic analyses show fewer activated eosinophils and mast cells in osteoarthritic knees than in healthy ones,^[^ ^22^, ^23^ ^]^ suggesting eosinophils as potential OA therapy targets.^[^ ^24^ ^]^ Recent studies show eosinophils play roles beyond allergy, including antigen presentation,^[^ ^25^, ^26^ ^]^ tissue repair via periostin,^[^ ^27^ ^]^ and osteoclast inhibition through the innate lymphoid cells type 2 (ILC2) axes,^[^ ^27^ ^]^ suggesting eosinophils may contribute to arthritis related to bone pathogenesis. Andreev found that eosinophils can inhibit osteoclast‐mediated bone resorption via eosinophil peroxidase (EPX) secretion, helping to maintain bone homeostasis and relieve rheumatoid arthritis (RA).^[^ ^28^ ^]^ While EPX regulates bone homeostasis,^[^ ^28^ ^]^ the role of eosinophils and their granule protein eosinophil major basic protein (EMBP),^[^ ^29^, ^30^, ^31^ ^]^ in cartilage and bone remodeling remains unexplored. Besides, IL‐5‐induced eosinophil increase in transgenic mice led to ectopic bone nodules in the spleen, disrupting organized osteogenesis in the tibia,^[^ ^32^ ^]^ indicating mixed osteogenic effects. Since TMJOA differs from peripheral RA in pathogenesis and structure, further research on eosinophils' role in TMJOA is needed.

Unlike the chronic autoimmune disease RA,^[^ ^33^ ^]^ TMJOA is a degenerative joint disorder specific to the TMJ, leading to gradual cartilage loss.^[^ ^1^, ^34^ ^]^ The TMJ, a synovial joint, forms from interactions between the mandibular condyle and squamosal bone, with a unique development of the articular disc from mesenchymal cells near the condyle's perichondrium.^[^ ^35^, ^36^, ^37^ ^]^ Unlike other joints covered by hyaline cartilage (type II collagen), TMJ has fibrocartilage (types I and II collagen), which influences its degeneration process.^[^ ^38^ ^]^ TMJ fibrocartilage uniquely contains fibrocartilage stem cells (FCSCs), providing superior healing and repair compared to other joints lacking such cells.^[^ ^39^, ^40^, ^41^ ^]^ In the rat mandibular condyle, fibrocartilage‐covered periosteum supports continuous growth and wound healing, unlike hyaline cartilage in long bones.^[^ ^42^ ^]^ Functionally, TMJ handles unique loading patterns from chewing, clenching, and bruxism, enabling adaptive remodeling.^[^ ^43^, ^44^ ^]^ Its fibrocartilage resists shear forces better, while hyaline cartilage in weight‐bearing joints suits compressive loads.^[^ ^45^, ^46^ ^]^ Treatment approaches also differ, as hyaluronic acid is less effective in restoring TMJ structure than in larger joints.^[^ ^47^, ^48^, ^49^, ^50^ ^]^ These unique TMJ properties underscore the need to investigate eosinophils' role in TMJOA. The crosstalk between synovial responses and cartilage degradation is key in driving the development of TMJOA. The synovium produces pro‐inflammatory mediators and chemokines that drive immune cell recruitment, angiogenesis, and cartilage degradation.^[^ ^51^, ^52^ ^]^ In turn, molecular fragments from degraded cartilage stimulate further inflammatory responses, exacerbating synovitis.^[^ ^53^ ^]^ Previous studies have largely overlooked the role of EOS in the crucial cellular crosstalk between synovium and cartilage in TMJOA. Given the complexity and importance of these cellular interactions, understanding crosstalk between synovium and cartilage driven by EOS is critical to uncover the pathological mechanisms of TMJ diseases. Therefore, we focus on the interactions between EOS, synovium, and cartilage in TMJ and their effects on TMJOA progression, focusing on the exploration of crucial target molecules, aiming to provide new ideas and strategies for the treatment of TMJ‐related diseases.

Therefore, in this study, based on the phenomenon of the difference in EOS number in TMJOA synovial fluid from healthy subjects and TMJOA subjects, we explored the effects of EOS on TMJOA and its potential mechanisms by establishing an OVA‐induced hyper‐eosinophilia asthma rat model and a rat model of antibody‐mediated eosinophil depletion in vivo. Additionally, we utilized an in vitro co‐culture system comprising synovial fibroblasts, chondrocytes, and eosinophils to explore these interactions further. Eosinophils in TMJOA reduced synovial inflammation, cartilage degradation, and subchondral bone resorption. The mechanism involves eosinophils stimulating increased Lumican secretion in the TMJ synovium, which negatively regulates the transforming growth factor β2 (TGF‐β) signaling pathway. Lumican, through its interaction with Annexin A1 (Anxa1) in chondrocytes, further inhibits the expression of TGFβ2 and the phosphorylation of Smad2/3. This regulatory process provides additional insight into the modulation of the crosstalk between synovial fibroblasts and chondrocytes, contributing to the inhibition of TMJOA progression, indicating eosinophils’ role and mechanism in promoting TMJOA resolution, providing new ideas and strategies for the therapy of TMJ‐related diseases.

## 2 Results

### 2.1 The Number of Eosinophils in Joint Synovial Fluid in TMJOA Patients is Significantly Lower than that in Healthy Individuals

**Figure 1:**
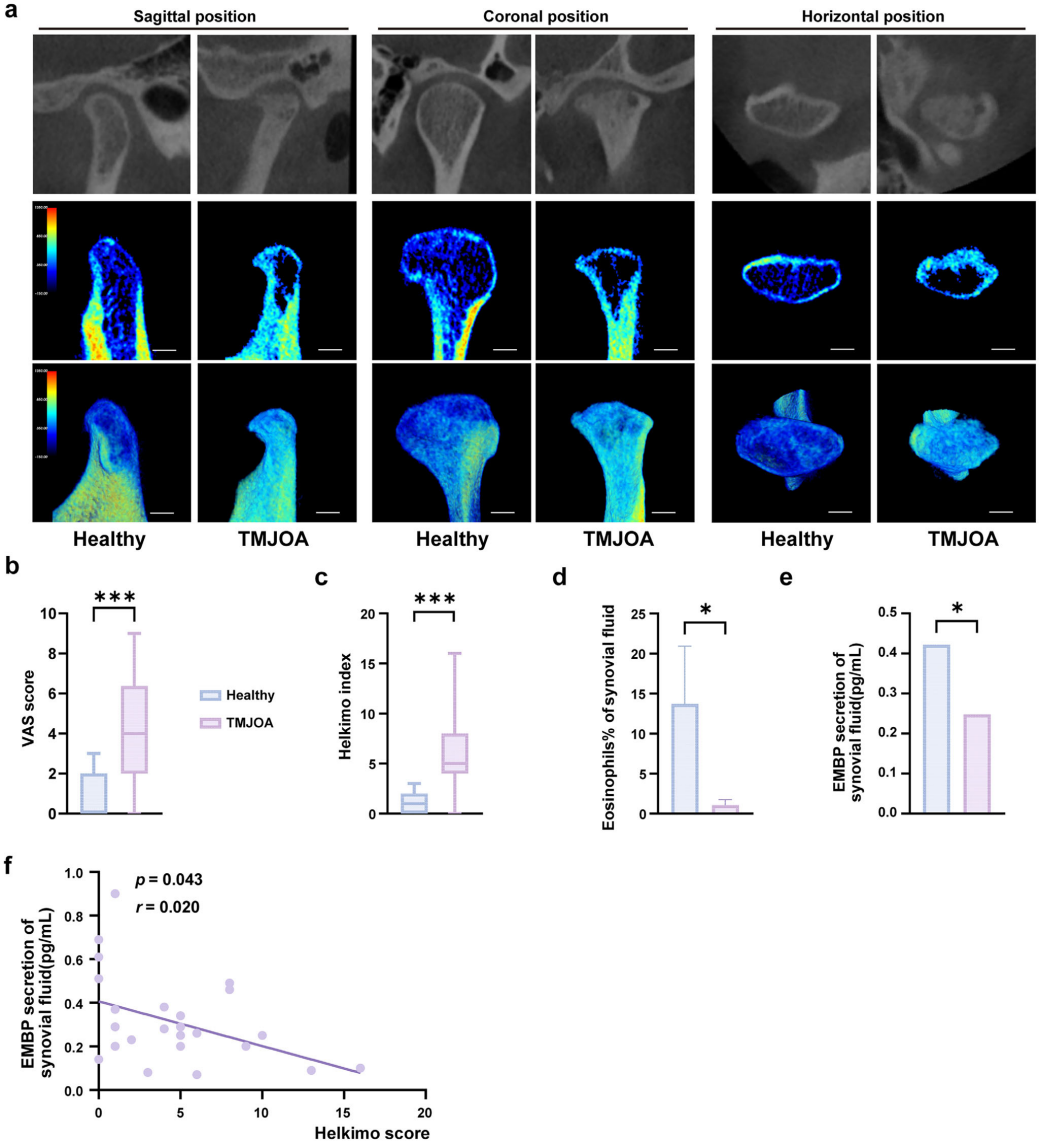
The number of eosinophils in joint synovial fluid in TMJOA patients was significantly lower than that in healthy controls. a) Sagittal, coronal, and horizontal CBCT cross‐sections and reconstruction images of TMJ showed significant cortical bone destruction, sclerosis, and bone erosion in the condyle of the TMJOA group compared with the control group. Scale bar, 2 mm. b,c) VAS score, and Helkimo clinical dysfunction index analysis of healthy group and TMJOA group. There were significant statistical differences between the two groups. n (Healthy) = 13 and n (TMJOA) = 20 for VAS score analysis. n (Healthy) = 13 and n (TMJOA) = 24 for Helkimo clinical dysfunction index analysis. d) The automatic count of eosinophils in the joint cavity synovial fluid using the whole blood automatic analyzer. n (Healthy) = 6 and n (TMJOA) = 9. e) The EMBP secretion of the joint cavity synovial fluid using ELISA detection. n (Healthy) = 7 and n (TMJOA) = 18. f) The correlation analysis of EMBP secretion in the joint cavity synovial fluid and Helkimo clinical dysfunction index. n (Healthy) = 37 and n (TMJOA) = 26. Statistical test: b) Mann‐Whitney test. c–e) Unpaired T‐test. f) Pearson correlation coefficients analysis. *P < 0.05, ***P < 0.001.

To investigate whether there are differences in eosinophils in temporomandibular joint synovial fluid between healthy individuals and TMJOA patients, a single‐center clinical study was conducted to investigate eosinophils differences in the TMJ synovial fluid. The study collected TMJ synovial fluid, cone‐beam computed tomography (CBCT) imaging data, visual analog scale pain scores (VAS), and Helkimo clinical dysfunction index (Di). Significant cortical bone destruction and sclerosis were observed in the diseased group (**Figure** **1**a). Besides, as shown in Figure 1b,c and Tables S1 and S2 (Supporting Information), there were significant differences in VAS scores (Figure 1b, Table S1, Supporting Information) and Di values (Figure 1c, Table S2, Supporting Information) between the two groups, indicating that the disease group experienced more severe TMJ pain and more pronounced jaw motion restriction. Furthermore, we explored the difference in eosinophils quantity in the synovial fluid of the TMJ cavity between the patients with TMJOA and the healthy population. The counts of EOS in TMJOA patients’ synovial fluid were nearly undetectable, while the healthy had a detectable and significantly higher proportion of eosinophils in white blood cells (Figure 1d). EMBP measured by ELISA was also dramatically lower in the TMJOA group (Figure 1e), aligning with the results of eosinophil counts. Additionally, correlation analysis showed a weak negative correlation (*r* = 0.2) between EMBP levels and Di values (Figure 1f). Di assesses TMJ function based on mandibular movement, joint impairments (e.g., clicking, crepitus), and pain, categorizing dysfunction as no (0), mild (1–4), moderate (5–9), or severe (≥10).^[^ ^54^, ^55^ ^]^ In this study, TMJOA patients had a median Di of 5 (4–8) (Table S2, Supporting Information), indicating moderate dysfunction. The correlation between EMBP levels and Di values highlights EMBP's potential as a biomarker. Future studies should investigate the mechanisms linking EMBP to TMJOA dysfunction, including its correlation with specific functional impairments encompassed by Di, and explore the therapeutic potential of EMBP‐targeted interventions. Therefore, we observed a reduction in the number of eosinophils in the TMJOA patients’ synovial fluid, suggesting that eosinophils may play an essential protective role in maintaining the homeostasis of the TMJ.

### 2.2 OVA‐Induced Hyper‐Eosinophilia Asthma causes Regression of Synovial, Cartilage, and Subchondral Bone Symptoms in TMJOA

**Figure 2:**
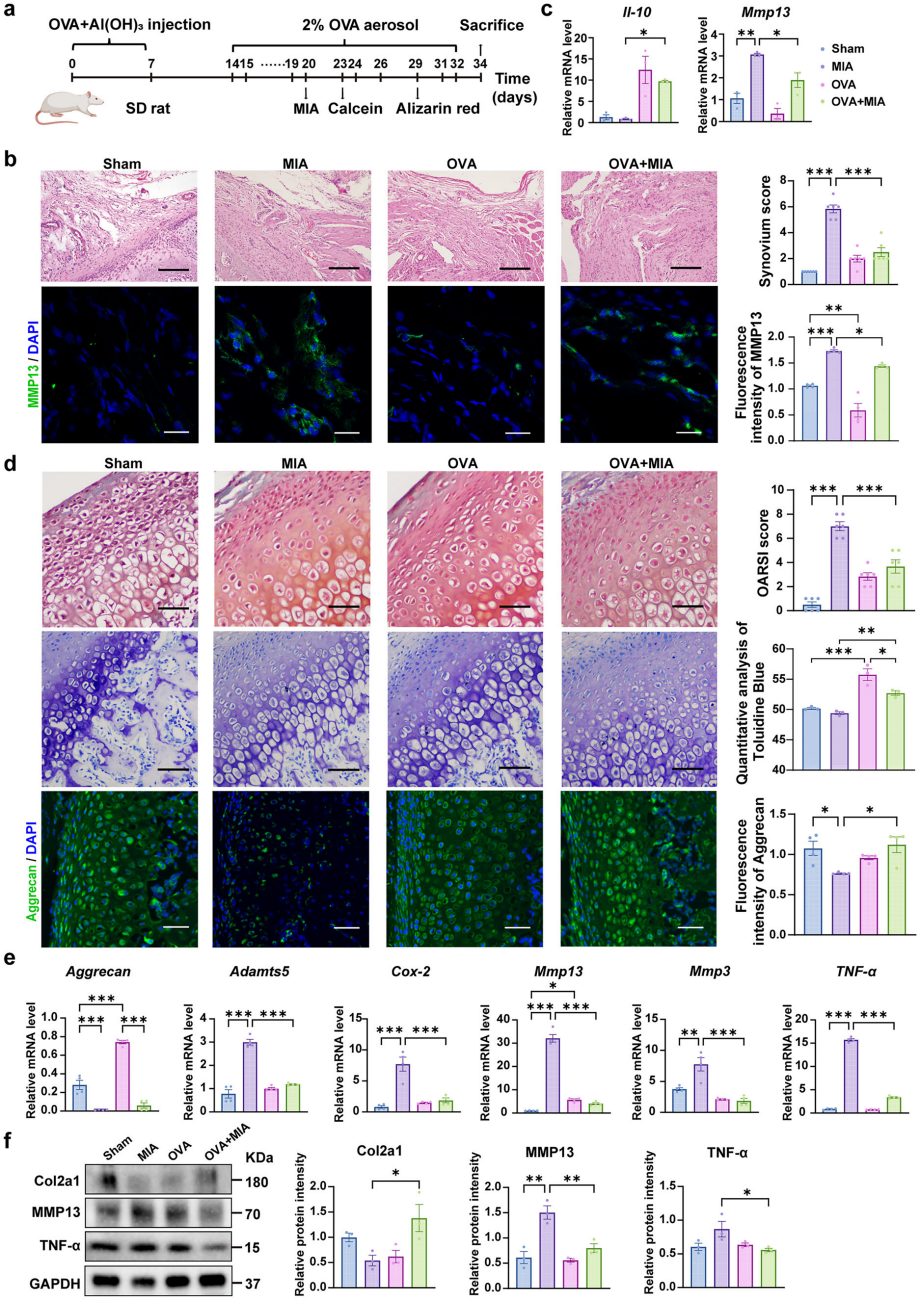
OVA‐induced hyper‐eosinophilia asthma causes regression of synovial cartilage symptoms of the temporomandibular joint osteoarthritis. a) Experimental outline of OVA‐induced allergic hyper‐eosinophilia asthma and MIA‐induced temporomandibular osteoarthritis in SD rats. b) H&E staining of rat synovium and synovium scores were measured. Scale bar, 100 µm. MMP13 expression on synovial tissue was detected by immunofluorescence staining by confocal laser scanning microscopy (CLSM), and fluorescence intensity was analyzed. Green, MMP13; Blue, DAPI. Scale bar, 10 µm. c) Quantitative real‐time PCR (qRT‐PCR) analysis of the indicated genes of rat synovial tissue. d) Safranin O‐fast green staining of temporomandibular joint and analysis of OARSI score. Scale bar, 50 µm. Toluidine blue‐stained cartilage of rats and quantitative analysis. Scale bar, 50 µm. Aggrecan expression on cartilage tissue was detected by immunofluorescence staining by CLSM, and Fluorescence intensity was analyzed. Scale bar, 10 µm. e) qRT‐PCR analysis of the indicated genes of rat cartilage tissue. f) Western blots and analysis of Col2a1, MMP13, TNF‐α in total protein extracts of cartilage tissues from rats. GAPDH is a housekeeping gene used as a loading control. Statistical test: One‐way ANOVA Dunnett's test. *P < 0.05, **P < 0.01, ***P < 0.001.

An ovalbumin (OVA)‐induced hyper‐eosinophilia asthma rat model was established to assess whether allergic eosinophils could mitigate TMJOA in rats. After successful asthma induction, TMJOA rats were induced (**Figure** **2**a). In the OVA group, there was evident infiltration of inflammatory cells in the lungs and increased eosinophils compared to the Sham group. The OVA group exhibited increased eosinophil‐specific stained cells compared to the Sham group, with flow cytometry confirming an elevated cell count. The eosinophil level of peripheral blood was also higher (Figure S1a–d, Supporting Information).

Compared to the Sham group, the sodium iodoacetate (MIA) group exhibited significant differences in condylar shape and vascularization. Specifically, the condyle in the MIA group showed pitted erosive resorption, flattening, and a reduction in head height. The condylar cross‐sectional area was significantly smaller, with reduced anteroposterior dimensions, resulting in a broader and flattened oval shape. Furthermore, vascularization on the condylar surface was notably increased, and the surface appeared reddish in color.

In contrast, the OVA+MIA group demonstrated opposite changes. In this group, condylar resorption was reduced, the surface was smoother, and cortical bone continuity improved. The condylar height partially recovered compared to the MIA group, and the cross‐sectional area increased, displaying an elongated oval shape. Additionally, vascularization on the surface decreased, and the condyle exhibited a lighter reddish color (Figure S2a, Supporting Information), indicating that OVA is conducive to alleviating the continuous destruction and bone resorption of the condylar cortex by TMJOA. Synovitis is the earliest phenotype of TMJOA. In the MIA group, thickened synovial tissue, increased inflammatory cells, and vascular proliferation were observed compared with the Sham group, but OVA treatment reduced synovial proliferation, vascular dilation, and histopathological scores. MMP13 immunofluorescence analysis in synovium revealed that the expression of MMP13 in the MIA group was significantly higher compared to the Sham group (Figure 2b). Conversely, the intervention with OVA in the OVA+MIA group led to a reduction in MMP13 expression. In addition, OVA also reduced *Mmp13* expression and increased *Il‐10* mRNA expression levels in synovial tissue (Figure 2c). Compared with the MIA group, OVA+MIA group sharply increased the ratio of fibrocartilage (FC) / calcified cartilage (CC) (Figure S2b, Supporting Information), and elevated osteoarthritis research society international (OARSI) scores (Figure 2d). As shown in Toluidine blue staining, the cartilage was significantly upregulated in the OVA+MIA group compared to the MIA group (Figure 2d). This result was consistent with the immunofluorescence and qRT‐PCR result of Aggrecan in rat cartilage (Figure 2e). It showed that, compared with the MIA group, the mRNA levels of *Adamts5*, *Cox‐2*, *Mmp13*, *Mmp3*, and *Tnf‐α*, were prominently downregulated in the OVA+MIA group (Figure 2e). Additionally, OVA treatment in OVA+MIA group downregulated the protein levels of MMP13 and TNF‐α and upregulated the level of Col2a1 compared with MIA group in rat cartilage (Figure 2f). These findings suggested that OVA‐induced asthma attenuates TMJOA‐related synovitis and cartilage degradation, contributing to symptom relief in TMJOA.

**Figure 3:**
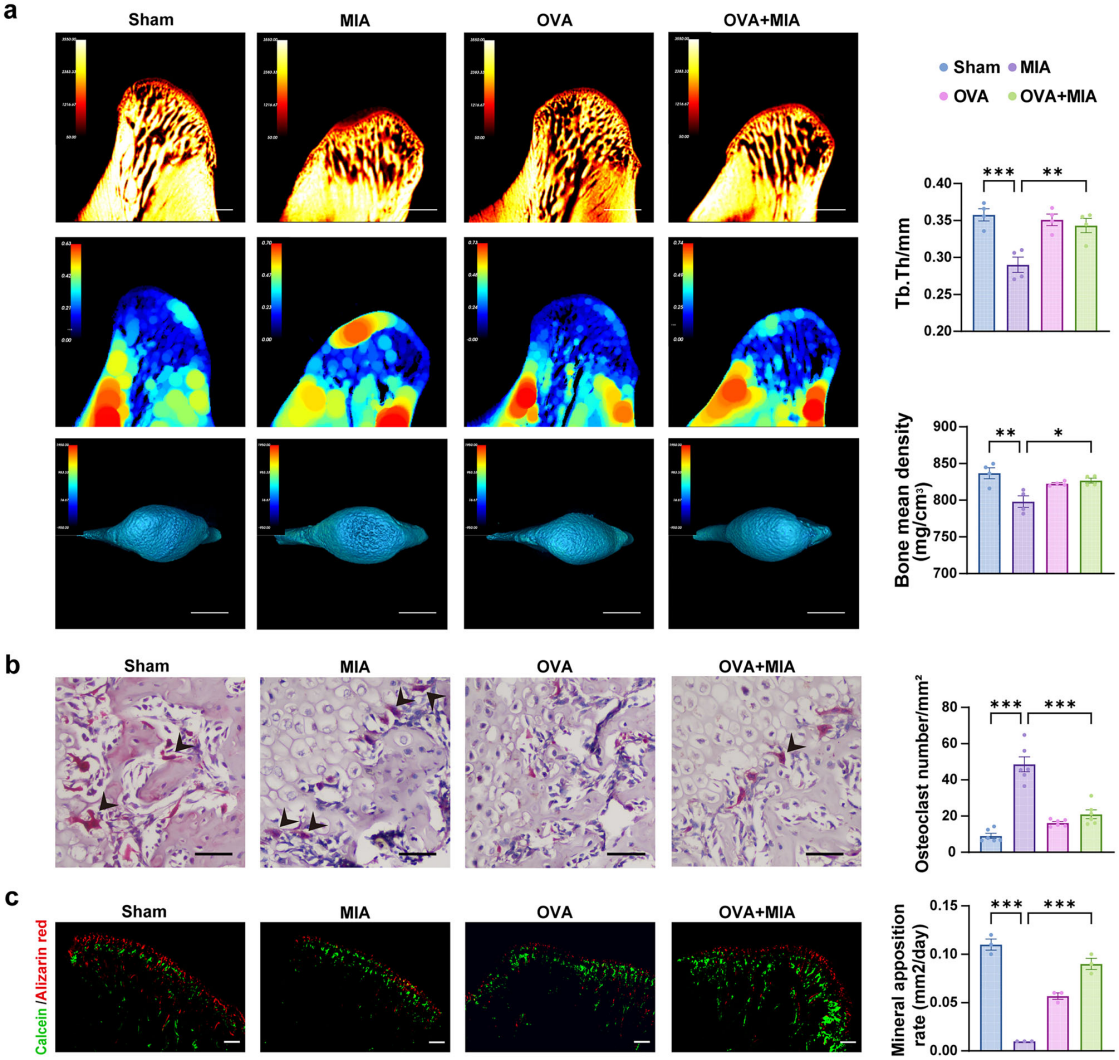
OVA‐induced hyper‐eosinophilia asthma reduced subchondral bone destruction caused by TMJOA. a Horizontal, sagittal, and 3D reconstruction of the sample by micro‐computed tomography and statistical analysis of parameters related to bone deterioration with micro‐CT. Scale bar, 1 mm. b TRAP staining and quantification of TRAP‐positive polynucleated (≥3 nuclei) osteoclast number per tissue area in subchondral bone of rats. The black arrows indicated TRAP‐positive polynucleated. Scale bar, 50 µm. **c** Calcein (green) and alizarin red (red) double‐labeled representative images of the subchondral bone region. Analysis of mineral apposition of subchondral bone in successive frozen sections (30 µm thick) in rats. Scale bar, 50 µm. Statistical test: One‐way ANOVA Dunnett's test. *P < 0.05, **P < 0.01, ***P < 0.001.

Micro‐CT was performed to reflect the morphological changes of the subchondral bone of the condyle from three dimensions. The 3D reconstruction images illustrated that the condylar height significantly decreased in the MIA group compared to the Sham group, with surface pitting and increased bone destruction. After OVA treatment, the OVA+MIA group exhibited a notable recovery in bone height and some restoration of cortical continuity compared to the MIA group (**Figure** **3**a). The results showed that the trabecular thickness (Tb.Th) and bone mineral density in the MIA group was significantly lower than that in the Sham group (Figure 3a), with trabecular separation (Tb.Sp) increased (Figure S2c, Supporting Information). In the OVA+MIA group, Tb.Th increased by 36% ± 8.5%, and bone mineral density increased by 3.6% ± 1.0% compared with the MIA group (Figure 3a). The number of tartrate‐resistant acid phosphatase (TRAP)‐positive cells in subchondral bone was apparently increased in MIA rats compared with Sham rats. The percentage of TRAP+ cells was significantly reduced in subchondral bone marrow after OVA induction in OVA+MIA rats (Figure 3b). Histomorphometry was performed by calcein and alizarin red. The rate of mineral apposition (MAR, mm^2^/day) was significantly raised in the OVA+MIA group compared to the MIA group (Figure 3c). Taken together, it indicates that OVA can rescue the abnormal morphological structure and inhibit subchondral bone resorption of the condyle in TMJOA.

### 2.3 Eosinophils are the Main Effector Cells Driving Asthma‐Induced TMJOA Alleviation

**Figure 4:**
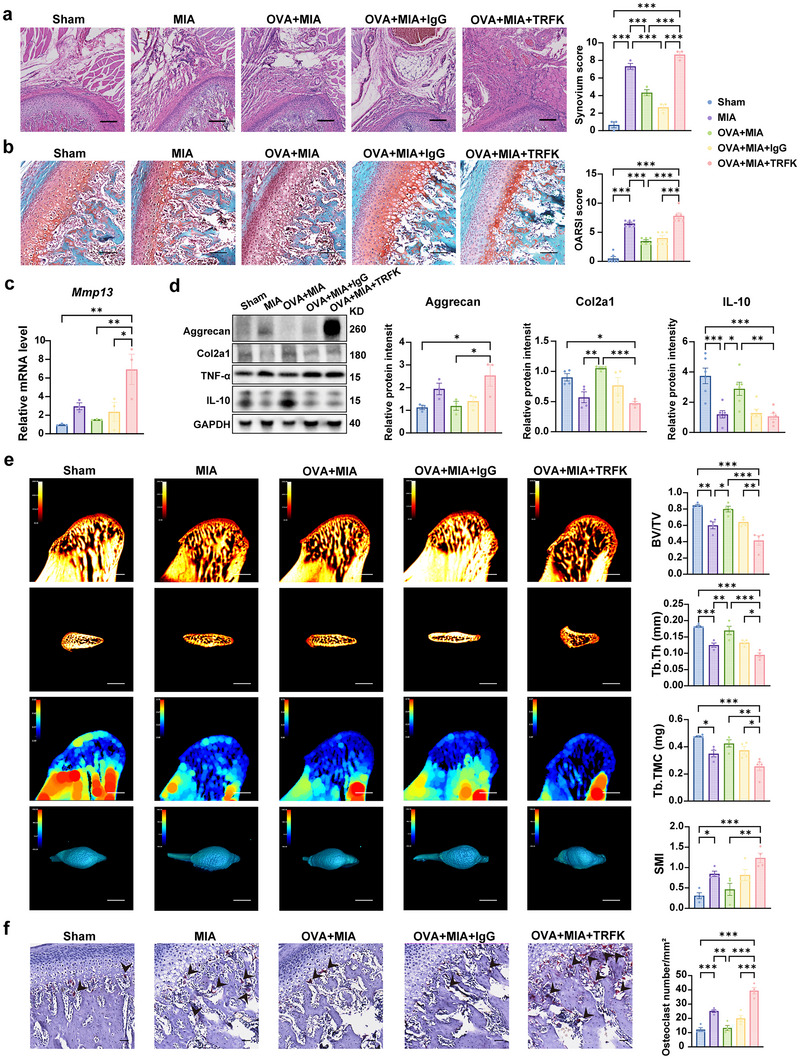
Eosinophils depletion led to worsening of TMJOA symptoms. a) H&E staining of rat synovium and synovium scores were measured. b) Safranin O‐fast green staining of temporomandibular joint and analysis of OARSI score. Scale bar, 50 µm. c) qRT‐PCR analysis of *Mmp13* of rat synovial tissue. d) Western blots and analysis of Aggrecan, Col2a1, IL‐10 in total protein extracts of synovium tissues from rats. GAPDH is a housekeeping gene used as a loading control. e) Horizontal, sagittal, and 3D reconstruction of the sample by micro‐computed tomography and statistical analysis of BV/TV, Tb.Th, TB.TMC and SMI. Scale bar, 1 mm. f) TRAP staining of subchondral bone in rats. The black arrows indicated TRAP‐positive polynucleated. Quantification of osteoclast number per cubic millimeter was analyzed. Scale bar, 100 µm. Statistical test: One‐way ANOVA Dunnett's test. *P < 0.05, **P < 0.01, ***P < 0.001.

IL‐5 is essential for eosinophil stimulation, differentiation, and maturation. To further validate the role of eosinophils in the asthma‐induced TMJOA alleviation, we depleted these cells using an anti‐IL‐5 antibody (TRFK5) and observed changes in the synovium, cartilage, and subchondral bone, while Sham rats received an irrelevant immunoglobulin G (IgG) control (Figure S3a, Supporting Information). Eosinophils were effectively depleted from the lung and blood (Figure S3b–d, Supporting Information). Notably, eosinophil‐deficient rats developed more severe TMJOA that persisted even after asthma induction, unlike the IgG rats, where OVA treatment reduced synovium and cartilage inflammation as well as bone erosion. Significant thickening of the synovium increased inflammatory cell infiltration, and vascularization were observed in the EOS depletion group (OVA+MIA+TRFK) with a higher pathological score compared to the OVA+MIA+IgG group without IL‐5 neutralizing antibody (**Figure** **4**a). EOS depletion reduced cartilage thickness, and disorganized chondrocyte arrangement with a higher OARSI score (Figure 4b) and a lower ratio of FC/CC (Figure S3e, Supporting Information) was exhibited in the OVA+MIA+TRFK group compared with OVA+MIA+IgG group. Toluidine blue staining showed more severe cartilage matrix loss in the anterior condyle (Figure S3f, Supporting Information) in the OVA+MIA+TRFK group compared with the OVA+MIA+IgG group, indicating that EOS deficiency exacerbated cartilage degradation. Consistently, eosinophil depletion led to increased *Mmp13* mRNA expression (Figure 4c). Aggrecan protein expression levels were increased, leading to hypertrophic synovial appearance and a reduction in the cells' differentiation potential. Protein expression of Aggrecan, Col2a1, and IL‐10 was decreased in synovium in the OVA+MIA+TRFK group compared with the OVA+MIA+IgG group (Figure 4d). Altogether; those findings suggest that EOS deficiency exacerbates abnormal synovial proliferation and inflammation while accelerating cartilage degradation.

Micro‐CT analysis revealed more severe subchondral bone damage and trabecular remodeling in the OVA+MIA+TRFK group compared with the OVA+MIA+IgG group. Critical bone microarchitecture parameters further confirmed this deterioration. Specifically, the BV/TV and Tb.Th, which represents the proportion of bone volume to total volume and the thickness of trabeculae, respectively, were significantly reduced, indicating substantial bone loss and structural weakening. The tissue mineral content (TMC), which reflects the mineralized bone content in milligrams, was also lower in the TRFK group, further confirming impaired mineralization and bone quality. The structural model index (SMI) indicated a shift from plate‐like to rod‐like trabecular structures, signifying diminished structural integrity and load‐bearing capacity (Figure 4e). Additionally, elevated BS/BV and Tb.Sp highlighted increased porosity and greater spacing between trabeculae, while changes in degree of anisotropy (DA), a measure of trabecular alignment, suggested disrupted microarchitectural orientation (Figure S3g, Supporting Information). Moreover, TRFK‐treated rats showed increased osteoclast number compared with the IgG‐treated group (Figure 4f). This indicates that eosinophils are crucial for mitigating subchondral bone erosion in OVA‐induced eosinophilic asthma.

### 2.4 Eosinophils Negatively Regulate the TGF‐β Signaling Pathway

**Figure 5:**
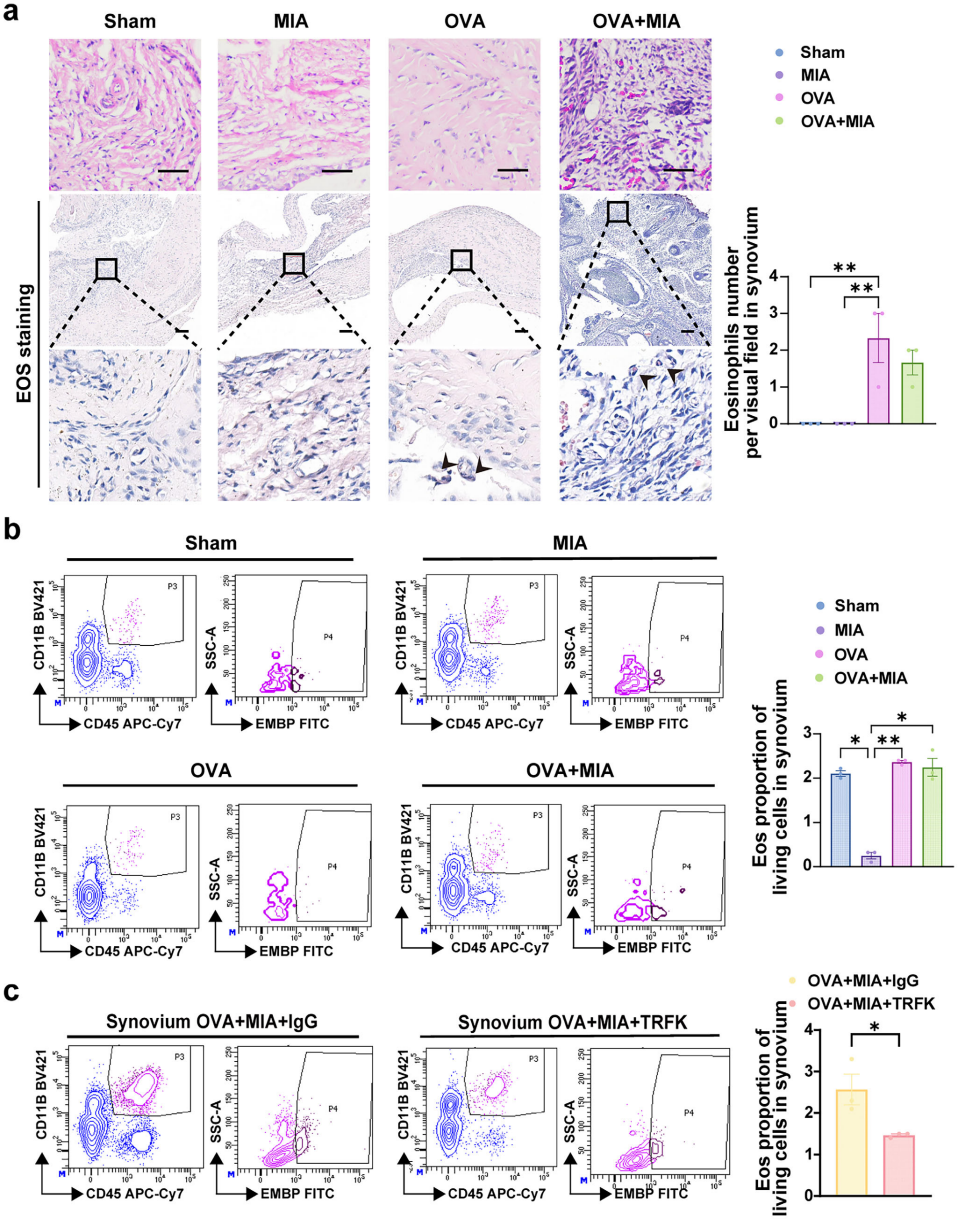
Eosinophils are the main effector cells driving asthma‐induced resolution of TMJOA by OVA. a) H&E and EOS staining of rat synovium and EOS per visual field were measured. The black arrows indicated EOS. Scale bar, 100 µm. b) Quantification of EOS (CD11b^+^CD45^+^EMBP^+^cells) in the synovium of rats with OVA/MIA treatment analyzed by flow cytometry. c) Quantification of EOS (CD11b^+^CD45^+^EMBP^+^cells) in the synovium of rats with OVA/MIA/TRFK or IgG treatment analyzed by flow cytometry. Statistical test: a‐b, One‐way ANOVA Dunnett's test. c) Unpaired T‐test. *P < 0.05, **P < 0.01.

Eosinophils are primarily distributed in the synovial tissue of joints, and there is a notable difference in the distribution of eosinophil infiltration between normal tissues and the synovium of osteoarthritic tissues.^[^ ^22^ ^]^ Therefore, to investigate the changes in eosinophil distribution in the TMJ synovium following OVA induction, we employed specific eosinophil staining and flow cytometry to further assess EOS alterations in synovial eosinophils. Results showed an increase in eosin‐stained eosinophils in synovial tissue after OVA induction (**Figure** **5**a), confirmed by flow cytometry, which revealed more evident recruitment of CD45^+^CD11B^+^EMBP^+^ eosinophils in the OVA group compared to the Sham group (Figure 5b). Following anti‐IL‐5 TRFK treatment to deplete eosinophils, EOS content in synovial tissue was significantly reduced compared to the IgG control group (Figure 5c). These findings suggested that the elevated local recruitment of eosinophils in the synovium may contribute to TMJOA remission after OVA induction.

**Figure 6:**
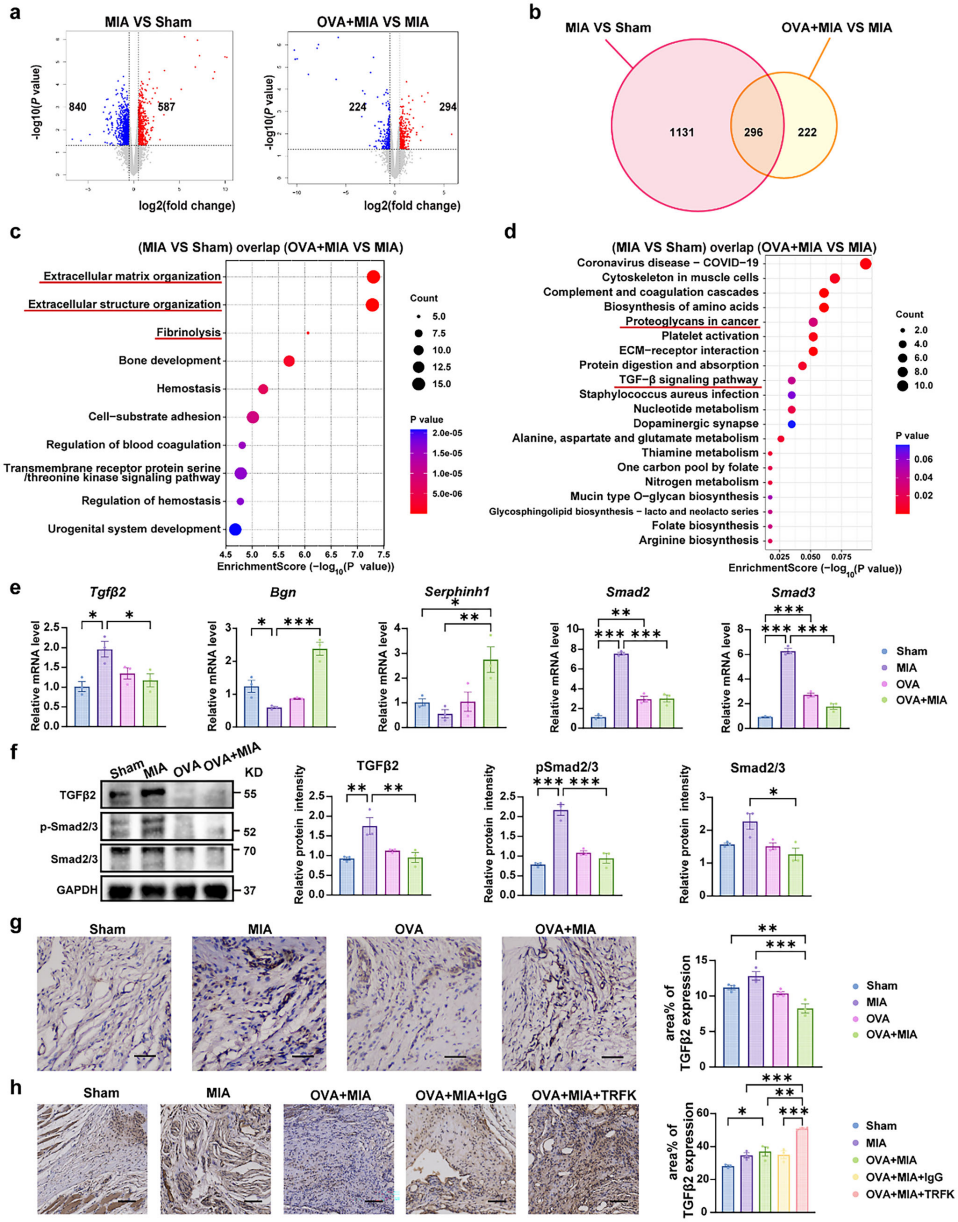
RNA‐seq analysis reveals that EOS negatively regulates the TGF‐β signaling pathway. a) Volcano plot visualizing DEGs between the MIA group and Sham group and between the OVA+MIA and MIA group. The *P* value <0.05 was used as a threshold to determine the significance of DEGs. Red dots represent upregulated DEGs, blue dots represent downregulated DEGs, and gray dots indicate transcripts that did not change significantly between the two groups. b) Overlapping genes of DEGs from (MIA VS Sham) and DEGs from (OVA+MIA VS MIA). c,d) GO and KEGG enrichment analysis of overlapping genes shown in Figure 6b. The darker the color, the smaller the q value. Bubble size indicates the DEG number. e) Relative expression levels of mRNAs (*Tgfβ2, Bgn, Serphrinh1, Smad2, and Smad3*) normalized by the rat *Gapdh* gene in rats treated with OVA/MIA by qRT‐PCR analysis. f) Western blotting analysis of TGFβ2, total and phosphorylated forms of Smad2/3 in rats. GAPDH is a housekeeping gene used as a loading control. g) IHC analyses of TGFβ2 in OVA/MIA treatment rats compared with sham‐operated rats. h) IHC analyses of TGFβ2 in OVA/MIA/TRFK or IgG treatment rats compared with sham‐operated rats. Statistical test: One‐way ANOVA Dunnett's test. *P < 0.05, **P < 0.01, ***P < 0.001.

RNA sequencing analysis of synovial tissue was performed to explore further the molecular mechanisms by which OVA‐induced eosinophils in synovium alleviate TMJOA. A total of 1427 differentially expressed genes (DEGs) (840 upregulated genes and 587 downregulated genes) were identified between the MIA group and the Sham group, and 518 DEGs (224 upregulated genes and 294 downregulated genes) between the OVA+MIA group and the MIA group (**Figure** **6**a). Among all DEGs, 296 genes were found to be present simultaneously within each respective set of overlapping differentially expressed genes (Figure 6b). GO analysis of those 296 genes revealed the enrichment in biological process related to “extracellular matrix organization,” “extracellular structure organization” and “fibrinolysis,” which were associated with collagen assembly and organization (Figure 6c). KEGG analysis highlighted the “Proteoglycans in cancer” and “TGF−β signaling pathway” as significantly enriched in overlapping DEGs between the groups (Figure 6d).

To verify the RNA‐seq analysis, we performed qRT‐PCR to detect TGF‐β pathway‐related genes, including *Tgfβ2* and its downstream targets *Bgn*, *Serphinh1*, *Smad2*, and *Smad3* with RPKM > 30 that we reviewed in the literature.^[^ ^56^, ^57^ ^]^ It showed that OVA treatment downregulated the expression of *Tgfβ2, Smad2*, and *Smad3* and upregulated the *Bgn*, *Serphinh* in the synovium of TMJOA rats, suggesting the inhibition of TGF‐β signaling (Figure 6e). Consistently, compared to the MIA group, the protein expressions of TGFβ2 and Smad2/3 phosphorylation were dramatically downregulated in the OVA+MIA group in rat synovial tissue compared with MIA group (Figure 6f). It is noteworthy that immunohistochemistry experiment indicated the elevated TGFβ2 expression in the synovium of MIA rats, which was reversed after the OVA treatment in OVA+MIA group (Figure 6g). Moreover, compared with the OVA+MIA+IgG group, the treatment of eosinophil depletion sharply upregulated the protein level of synovial TGFβ2 in the OVA+MIA+TRFK group (Figure 6h). Collectively, these findings identify that OVA‐induced eosinophils alleviate TMJOA primarily by inhibiting the TGF‐β signaling pathway in synovial tissue.

### 2.5 Eosinophils impede the TGF‐β signaling pathway via the promotion of Lumican secretion in the synovium of TMJOA

**Figure 7:**
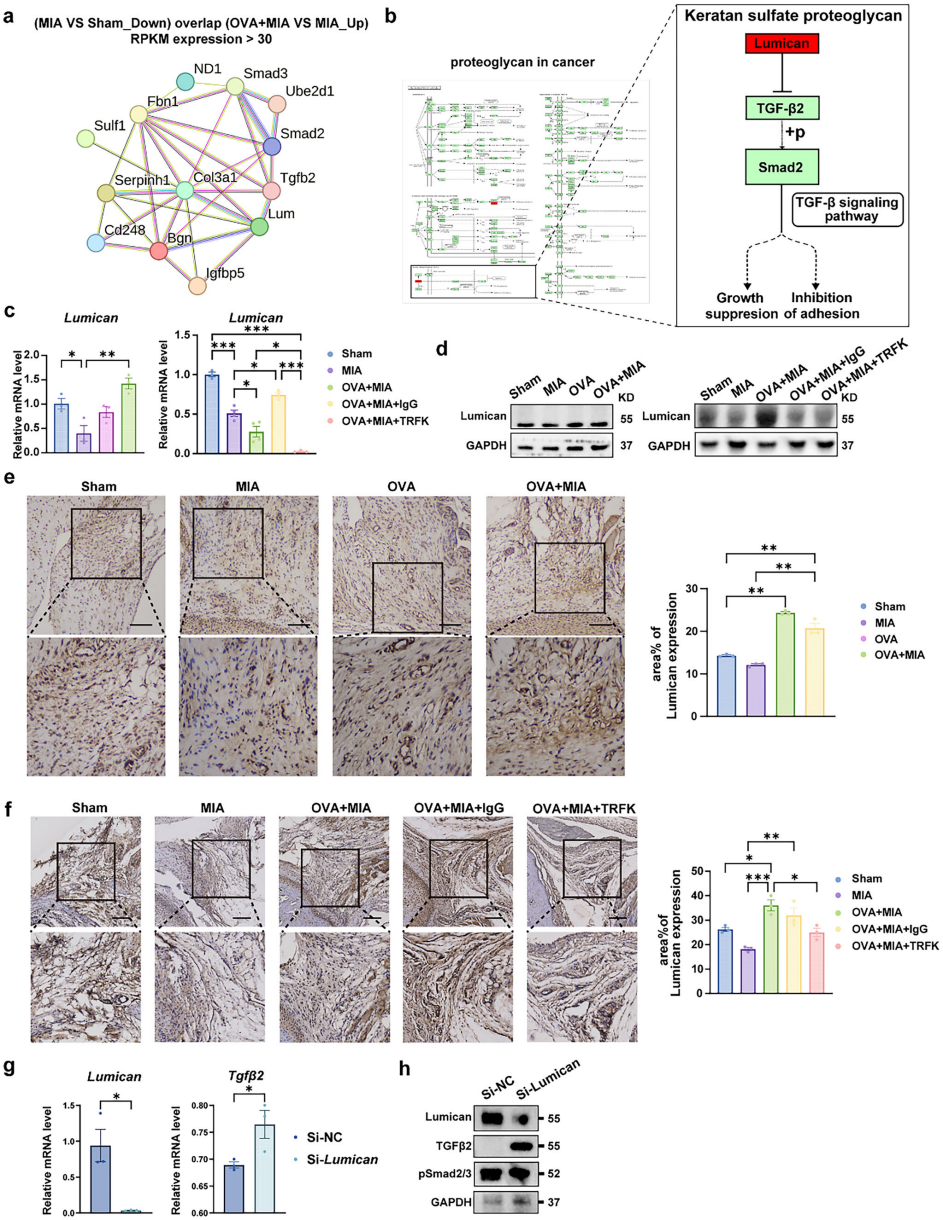
EOS decreased the expression of the TGF‐β signaling pathway by promoting the secretion of Lumican in synovial tissue. a) Protein‐protein interaction (PPI) was performed for the overlapping genes with more than 30 RPKM expression levels. The unconnected proteins were removed. b) Illustration of the proteoglycan in cancer pathway identified in the KEGG enrichment analysis, as presented in Figure 7a. The magnified section highlighted the signaling pathways associated with keratan sulfate proteoglycan within the proteoglycan in the cancer pathway, revealing the protein interaction between Lumican and TGFβ2. c) qRT‐PCR analysis of *Lumican* of rat synovial tissue. d) Western blotting analysis of Lumican in the synovium of rats. e) IHC analyses of Lumican in OVA/MIA treatment rats compared with sham‐operated rats. f) IHC analyses of Lumican in OVA/MIA/TRFK or IgG treatment rats compared with sham‐operated rats. Scale bar, 100 µm. g) Changes in mRNA expression levels of *Lumican*, *Tgfβ2* following *Lumican* knockdown in synovial fibroblasts using siRNA. h) Changes in protein expression levels of Lumican, TGFβ2, pSmad2/3 after *Lumican* knockdown in synovial fibroblasts using siRNA. Statistical test: One‐way ANOVA Dunnett's test. *P < 0.05, **P < 0.01, ***P < 0.001.

To further explore how eosinophils regulated the TGF‐β signaling pathway, we screened key target molecules through protein‐protein interaction (PPI) analysis. Disconnected nodes were excluded, identifying Lumican as a central hub protein with high connectivity among overlapping genes. Its strong association with TGF‐β2 and downstream Smad2/3 signaling in the PPI network and biological functions led to its selection as a key target for investigating synovium‐cartilage crosstalk (**Figure** **7**a). In addition, KEGG pathway analysis further confirmed that Lumican could regulate the TGF‐β signaling pathway via the keratan sulfate proteoglycan, which is associated with extracellular matrix in proteoglycan in cancer KEGG pathway, highlighting its potential role in TMJOA progression (Figure 7b). To confirm Lumican's role as a critical regulator of TGF‐β signaling, qRT‐PCR, and western blot were conducted on synovial tissues. The mRNA expression of *Lumican* was downregulated in the MIA group compared to the Sham group but obviously raised after OVA‐induced eosinophils recruitment (Figure 7c left). Conversely, the increased mRNA expression level of *Lumican* was hindered when eosinophils were eliminated by TRFK (Figure 7c right). The expression trend of the Lumican protein is consistent with that of its mRNA (Figure 7d). Similarly, immunohistochemical analysis showed the same trend, manifesting that the increased expression level of Lumican in OVA+MIA synovial tissue was reversed after the eosinophil deletion (Figure 7e,f). We utilized small interfering RNA (siRNA) to knock down *Lumican* in synovial fibroblasts (SFs) isolated and cultured in vitro experiments, further investigating the impact of *Lumican* silencing on TGFβ2 expression. Compared to SFs transfected with non‐targeting siRNA (Si‐NC‐SFs), knockdown of *Lumican* in synovial fibroblasts (Si‐*Lum‐*SFs) resulted in an increased mRNA expression of *Tgfβ2* (Figure 7g) accompanied by a corresponding elevation in its protein expression levels (Figure 7h). These data suggests that eosinophils induce Lumican secretion of the synovium, which inhibit the TGF‐β signaling pathway and Smad2/3 phosphorylation in synovial tissue, thereby attenuating synovial inflammation in TMJOA progression.

### 2.6 Eosinophils Alleviate Cartilage Degradation by Promoting the Lumican Secretion in the Synovial Tissue in TMJOA

**Figure 8:**
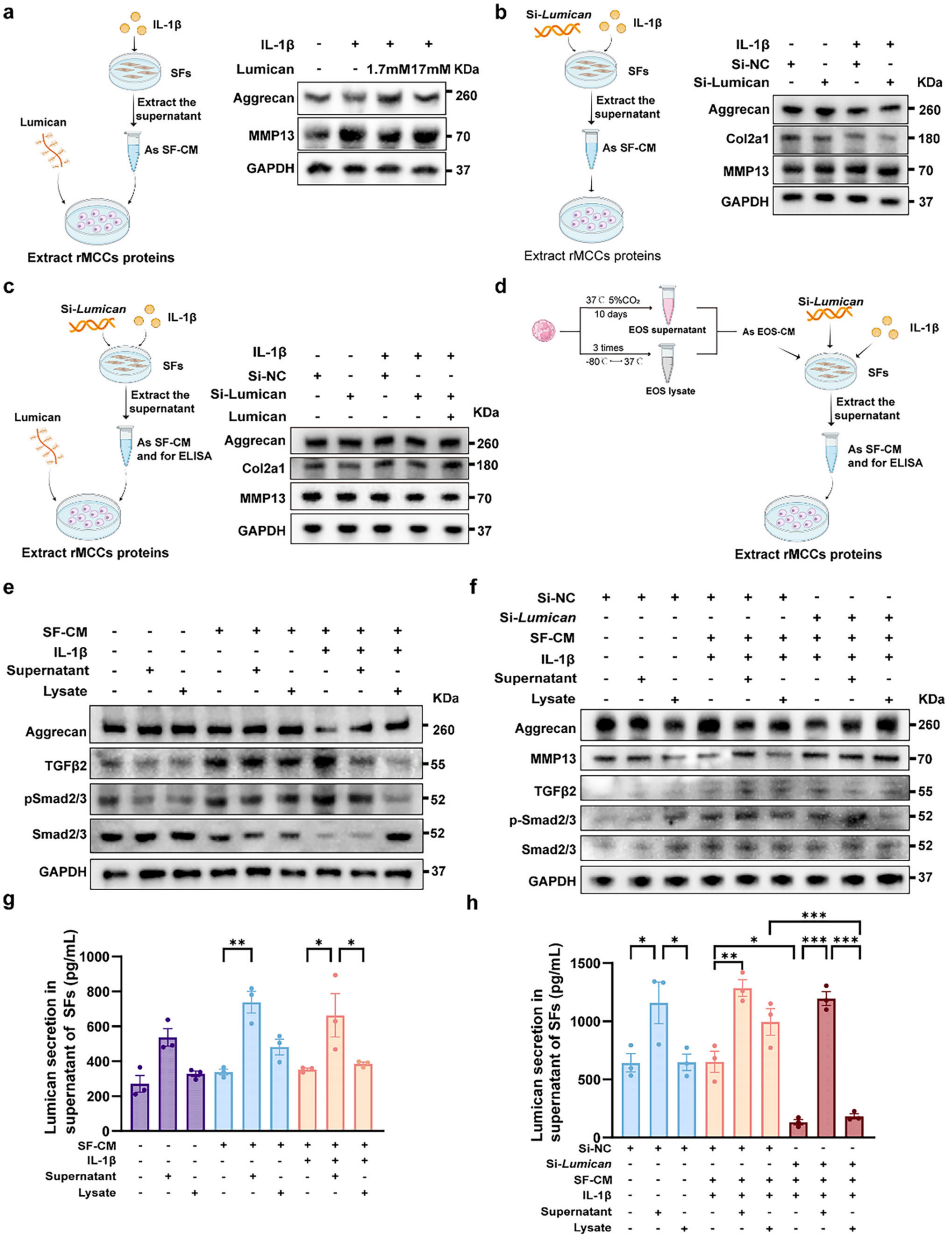
EOS alleviates condylar cartilage degradation by promoting the secretion of Lumican and reducing the expression of the TGF‐β signaling pathway in the synovial tissue. a) Strategies and western blot analysis in vitro for extracting rMCCs protein cultured with SFs supernatants for 12 h. SFs were treated with or without IL‐1β (5 ng mL^−1^) for 12 h. b) Strategies and western blot analysis extracting rMCCs protein cultured with transfected SFs (targeting *Lumican* and *Gapdh*) supernatants for 12 h. c) Strategies and western blot analysis for extracting rMCCs protein cultured with transfected SFs (targeting *Lumican* and *Gapdh*) supernatants for 12 h. Lumican was added to the culture medium of rMCCs. d) Strategies and western blot analysis for extracting rMCCs protein cultured with SFs supernatants (DMEM mixed with EOS supernatant or EOS lysate) for 12 h. e) Western Blotting analysis of Aggrecan, TGFβ2, pSmad2/3 and Smad2/3 for rMCCs with SFs supernatants cultured for 12 h. DMEM medium mixed with EOS supernatant or lysate was used as the conditioned medium based on Figure 8d for SFs culture. f) Western Blotting analysis of Aggrecan, MMP13, TGFβ2, pSmad2/3 and Smad2/3 for rMCCs with SFs (Si‐NC‐SFs or Si‐*Lum*‐SFs) supernatants cultured based on Figure 8d for 12 h. g) Conditional medium for SFs was collected to analyze Lumican secretion by ELISA. h) Conditional medium for SFs (Si‐NC‐SFs or Si‐*Lum*‐SFs) was collected to analyze Lumican secretion using ELISA. Statistical test: One‐way ANOVA Dunnett's test. *P < 0.05, **P < 0.01, ***P < 0.001. Some of the images were generated by biorender.com.

Lumican plays a critical role in the structural organization and function of the extracellular matrix.^[^ ^58^ ^]^ To investigate the role of Lumican in cartilage and its interaction with eosinophils, we established several co‐culture systems (detailed in the Methods section). First, we directly stimulated rat mandibular condylar chondrocytes (rMCCs) with recombinant rat Lumican (**Figure** **8**a). The results showed that the intervention of Lumican reversed the effect of supernatant of SFs treated by IL‐1β on rMCCs, evidently restoring Aggrecan expression and reducing the elevated levels of MMP13 in rMCCs (Figure 8a; Figure S4a, Supporting Information). Furthermore, we explored whether SFs influenced rMCCs by secreting Lumican. The supernatant from Si‐*Lumican*‐SFs (Si‐*Lum*‐SFs) resulted in the decreased expression of Aggrecan and Col2a1 and increased MMP13 in rMCCs (Figure 8b; Figure S4b, Supporting Information). The downregulated level of Col2a1 and the upregulated level of MMP13 in rMCCs was more significant after the treatment of the supernatant from Si‐*Lum*‐SFs compared with Si‐NC‐SFs treated by IL‐1β (Figure 8b; Figure S4b, Supporting Information). To further verify whether exogenous‐Lumican could salvage this effect, we applied exogenously supplemented Lumican (Figure 8c). After the application of Lumican to rMCCs, the expression of Aggrecan and Col2a1 was significantly increased and the expression of MMP13 was decreased considerably (Figure 8c; Figure S4c, Supporting Information), indicating the protective effect of SFs‐derived Lumican on the metabolic homeostasis of cartilage matrix.

After confirming that SFs protected rMCCs via Lumican secretion, we further investigated whether eosinophils were involved in the interaction between SFs and rMCCs (Figure 8d). We isolated and cultured bone marrow‐derived EOS. Giemsa staining showed that the nuclei of eosinophils were billobulated or multilobulated, and the cytoplasm was filled with a large number of eosinophilic granules stained red (Figure S4d, Supporting Information). CD45^+^CD11b^+^EMBP^+^ EOS accounted for 85% by flow cytometry analysis (Figure S4e, Supporting Information). First, we added EOS supernatant or lysate directly as a conditioned medium to rMCCs. It was observed that both the supernatant and lysate of EOS enhanced the protein expression of Aggrecan in rMCCs (Figure 8e; Figure S4f, Supporting Information), with EOS lysate showing a more pronounced effect. Stimulated SFs with the supernatant or lysate of EOS, followed by the collection of SFs supernatant as the conditioned medium for rMCCs. Compared to without EOS supernatant or lysate, EOS lysate similarly increased the protein expression of Aggrecan in rMCCs. Still, there was no TGFβ2 change (Figure 8e; Figure S4g, Supporting Information). Notably, inducing inflammation in SFs using IL‐1β, the EOS supernatant improved the expression of Aggrecan in rMCCs, and the protective effect of the EOS lysate on rMCCs was more prominent. Besides, both the EOS supernatant and lysate downregulated the expression of TGFβ2 and pSmad2/3 (Figure 8e; Figure S4f–I, Supporting Information).

To further elucidate the role of Lumican secretion by synovial cells as a critical mediating pathway by which EOS influences cartilage, we applied EOS supernatants or lysates to SFs with *Lumican* knockdown (Si‐*Lum‐*SF). We then collected conditioned media from these treated SFs and applied it to rMCCs (Figure 8d). As shown in Figure 8f, under IL‐1β stimulation, rMCCs exposed to Si‐*Lum*‐SFs conditioned media exhibited a significant reduction in Aggrecan expression and an increase in MMP13 expression compared to those treated with Si‐NC‐SFs. Additionally, there was an elevation in TGFβ2 expression and phosphorylation levels of Smad2/3 in the chondrocytes treated with Si‐*Lum*‐SFs. Furthermore, after adding EOS supernatant to Si‐*Lum*‐SFs, Aggrecan expression in chondrocytes increased, and TGFβ2 expression decreased, compared to Si‐*Lum*‐SFs alone. Moreover, the application of EOS lysates, rather than EOS supernatants, to Si‐*Lum*‐SFs resulted in a more pronounced increase in Aggrecan and a more significant decrease in TGFβ2, indicating that EOS lysates exert a more substantial protective effect on cartilage.

To further investigate whether changes in Lumican following the addition of EOS contributed to the observed alterations in rMCCs, we detected Lumican in the SFs medium using ELISA (Figure 8c,d,g,h). According to strategies in Figure 8c, when EOS supernatant or lysate was not added to SFs, we found no significant difference in the levels of Lumican between the EOS intervention group and the non‐intervention group (Figure 8c‐left, g‐purple columns). However, upon adding EOS supernatant to SFs, the conditioned medium containing the EOS supernatant raised Lumican levels significantly compared to the group without EOS intervention. Meanwhile, although the addition of EOS lysate increased Lumican content, the difference was not statistically significant (Figure 8c‐left, g‐blue columns). After adding IL‐1β stimulation, we similarly observed that the secretion of Lumican by SFs was significantly higher in the group treated with EOS supernatant compared to the non‐intervention group. Furthermore, when EOS lysate was added instead of EOS supernatant, a notable reduction in Lumican secretion by SFs was observed (Figure 8c‐left, g‐orange columns). After knocking down *Lumican* and inducing inflammation with IL‐1β in SFs, according to Figure 8d‐left, the Lumican levels in the Si‐*Lum*‐SFs medium without EOS supernatant or lysate addition were significantly lower than that in Si‐NC‐SFs. Notably, following the addition of the EOS supernatant, the Lumican content in Si‐*Lum*‐SFs exhibited more pronounced changes compared to the group without EOS intervention. Similarly, adding EOS lysate did not significantly increase Lumican levels in SFs (Figure 8d‐left, h‐brown columns). In summary, the EOS supernatant and EOS lysate contain Lumican, with no significant difference in the Lumican content. However, adding EOS supernatant to SF led to a substantial increase in Lumican secretion, indicating that EOS notably stimulates SFs to secrete Lumican.

### 2.7 Lumican Modulates the TGFβ2/Smad2/3 Signaling Pathway in Chondrocytes via Annexin A1 Binding, Mitigating the Progression of TMJOA

**Figure 9:**
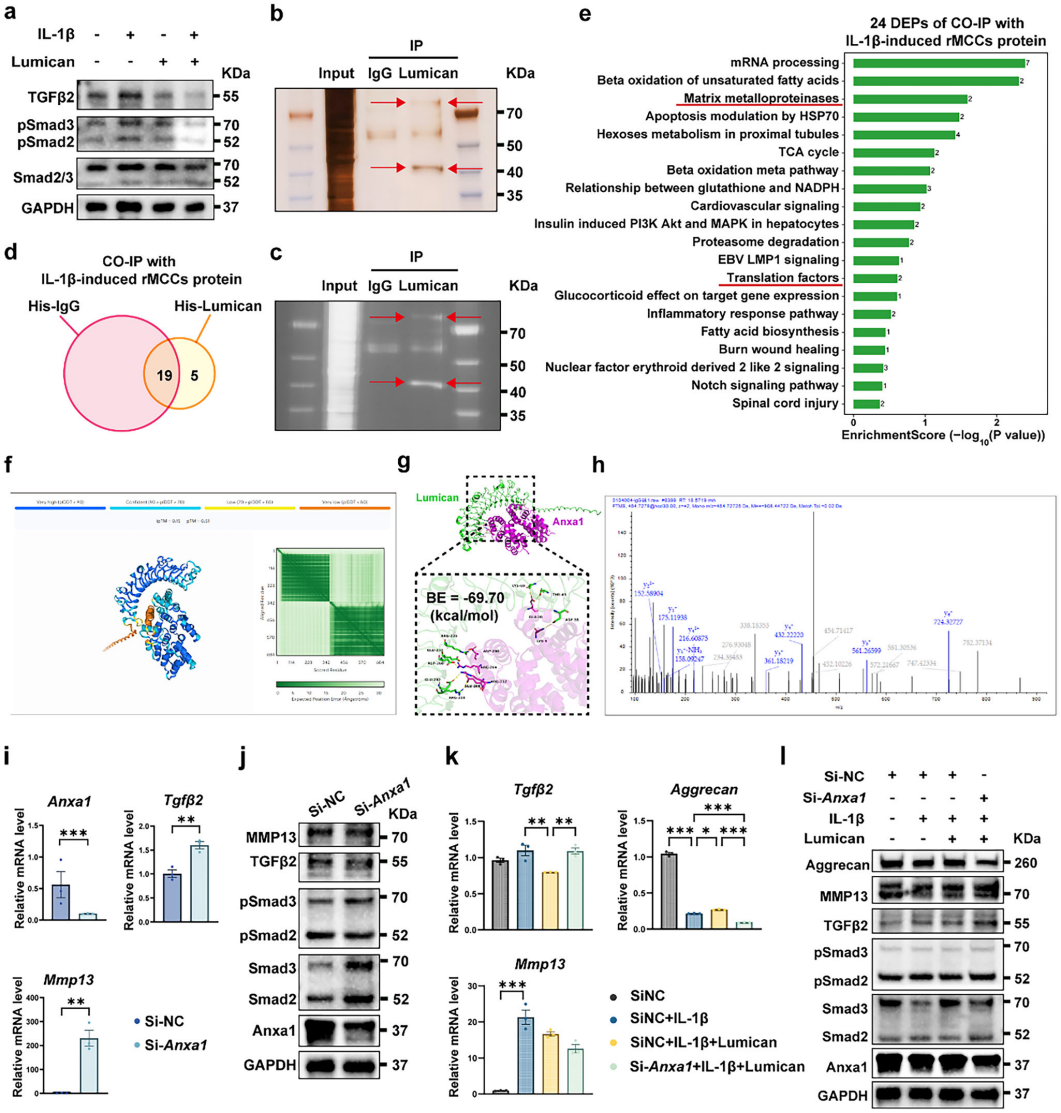
Lumican Modulates the TGFβ2/Smad2/3 Signaling Pathway in Chondrocytes via Annexin A1 Binding. a) IL‐1β and Lumican were added to chondrocytes, and proteins were extracted for Western blot analysis after 12 h. b) IL‐1β‐treated chondrocytes were subjected to CO‐IP with His‐McAb (marked as IgG) and His‐Lumican (marked as Lumican), followed by Western blot analysis and silver staining. The red arrow indicates a potential direct target of Lumican. c) The protein silver‐stained gel in panel b was subjected to colorimetric detection. d) Differentially expressed proteins (DEPs) that are uniquely expressed in the His‐Lumican group and differentially expressed between His‐McAb and His‐Lumican groups. e) Wikipathway enrichment analysis of the DEPs was shown in Figure 9d. f) Protein‐protein interaction prediction between Lumican and Anxa1 using the AlphaFold3 model. ipTM: predicted template modeling score; pTM: predicted template modeling score; pLDDT: predicted local distance difference test. g) Binding energy analysis and interaction residue prediction of Lumican (green) and Anxa1 (purple) using the AlphaFold3 model. BE: binding energy. h) Secondary mass spectrometry of Anxa1. i) Changes in mRNA expression levels of *Anxa1*, *Tgfβ2*, and *Mmp13* following *Anxa1* knockdown in rMCCs using siRNA. j) Changes in protein expression levels following Anxa1 knockdown in rMCCs using siRNA. k) rMCCs transfected with SiNC or Si‐Anxa1 were exposed to or not exposed to IL‐1β and Lumican for 12 h, and protein expression levels in chondrocytes were measured by western blot. Statistical test: i) Unpaired T‐test. k) One‐way ANOVA Dunnett's test. *P < 0.05, **P < 0.01, ***P < 0.001.

We further investigated the key mediator proteins involved in Lumican's inhibitory effect on the TGFβ2/Smad2/3 signaling pathway in chondrocytes. Initially, we added Lumican to IL‐1β‐induced inflammatory chondrocytes and observed that Lumican significantly reduced the phosphorylation of TGFβ2 and Smad2/3, which were elevated by IL‐1β. This finding confirmed that Lumican can inhibit the TGFβ2/Smad2/3 signaling pathway in inflammatory chondrocytes (**Figure** **9**a). To explore the potential binding receptor of Lumican that may regulate TGFβ2 in the inflammatory chondrocyte cellular membrane, we extracted membrane proteins from IL‐1β‐induced chondrocytes. We then performed co‐immunoprecipitation (CO‐IP) experiments using His‐tagged Mouse McAb (His‐IgG) as a control and His‐tagged recombinant Lumican protein (His‐Lumican) as the target. After protein extraction and denaturation, we used western blot for protein detection. Silver staining of the gel revealed that proteins interacting with Lumican were predominantly located in the 40–70 kDa region (Figure 9b,c). We subsequently excised the relevant region and subjected it to Liquid Chromatography‐Tandem Mass Spectrometry.

Among the detected proteins, we selected those that were either absent in the His‐IgG group but present in the His‐Lumican group or those that showed a 1.5‐fold higher expression in the His‐Lumican group compared to the His‐IgG group. A total of 24 differentially expressed proteins (DEPs) were identified, including 5 proteins exclusively expressed in the His‐Lumican group and 19 with differential expression between the two groups (Figure 9d). Pathway enrichment analysis of these 24 DEPs using Wikipathway revealed significant enrichment in biological processes related to “Matrix metalloproteinases” and “Translation factors” (Figure 9e). These results aligned with our previous GO and KEGG enrichment analysis from RNA sequencing (Figure 6c,d), indirectly validating our RNA sequencing findings. This suggests that the proteins involved in Lumican's action are related to the assembly of the collagen matrix.

To further identify the potential binding receptor of Lumican, we employed AlphaFold3 to generate a 3D protein model of Lumican based on its amino acid sequence. We then predicted the likelihood of interaction between Lumican and 24 DEPs, as well as the possible structural configurations of these protein complexes. Based on the AlphaFold3 protein interaction prediction results, the cellular localization of the proteins, and their reported roles in regulating TGFβ2,^[^ ^59^, ^60^ ^]^ we hypothesized that Anxa1 may be the protein that Lumican interacts with to modulate the TGFβ2/Smad2/3 signaling pathway. Anxa1 is a calcium‐dependent phospholipid‐binding protein predominantly found in the cytoplasm and cell membrane, involved in anti‐inflammatory responses, cell proliferation, and apoptosis, and plays a critical role in immune regulation and cell membrane repair.^[^ ^59^, ^60^ ^]^ In the AlphaFold3 prediction model, the interface predicted template modeling score (ipTM) was 0.15, and the predicted template modeling score (pTM) was 0.51. The predicted local distance difference test (pLDDT), which was between 50 to 70 for both proteins at the predicted interaction sites suggest a high probability of binding, with the interaction being relatively stable. The expected position error (EPE), represented by green grids, indicated that the structural prediction of this protein complex had relatively low EPE across most residues, suggesting that the positional information is accurate (Figure 9f). The interaction model indicated that the specific amino acids involved in the interaction may include lysine, arginine, glutamic acid, and others (Figure 9g) with −69.70 kcal mol^−1^ binding energy. Through these amino acid interactions, a binding interface between the two proteins is likely formed. The overall quality score of the model was moderate, suggesting that the predictions have a reasonable degree of reliability.

Further, we analyzed the secondary structure mass spectrum of the Anxa1 protein (Figure 9h) and compared it with the AlphaFold3 prediction results (Figure 9f,g). The secondary mass spectrum of Anxa1 revealed several high‐intensity signal peaks, particularly in the y‐ion series (e.g., y6‐, y5‐, y4‐), which are indicative of regions associated with the secondary structure. Stronger signals typically suggest that these regions are more stable and likely involved in the core folding of the protein (Figure 9h). Comparing the secondary mass spectrum of Anxa1 with the AlphaFold3 protein interaction prediction map, we observed that the high‐intensity signals (e.g., y4‐, y5‐ ions) (Figure 9h) corresponded well with the positions of the amino acid residues predicted to interact between Lumican and Anxa1 (Figure 9g,h). This consistency supports the notion that Lumican and Anxa1 indeed possess structurally stable regions that may be involved in their interaction.

We experimentally validated whether Lumican exerts its inhibitory effect on chondrocyte inflammatory responses by binding to Anxa1. Initially, we used siRNA to knock down *Anxa1* in rMCCs and examined changes in RNA and protein levels. The PCR results demonstrated a significant knockdown of *Anxa1* in rMCCs, with a marked reduction in its RNA expression. After *Anxa1* knockdown, RNA levels of *Tgfβ2* and *Mmp13* were significantly elevated in Si‐*Anxa1*‐rMCCs (Figure 9i). Western blot analysis also confirmed these findings, showing increased expression of TGFβ2 and pSmad2/3 in Si‐*Anxa1*‐rMCCs. To further investigate whether Anxa1 is a key binding protein for Lumican in regulating TGFβ2/Smad2/3 signaling pathway in IL‐1β‐induced rMCCs, we detected the effects of Lumican treatment in Si‐*Anxa1*‐rMCCs. Even with the addition of Lumican, rMCCs with *Anxa1* knockdown exhibited lower expression of Aggrecan compared to the Si‐NC‐rMCCs, while TGFβ2, MMP13, and phosphorylation levels of Smad2/3 were upregulated. These results suggest that the knockdown of *Anxa1* diminished Lumican's inhibitory effect on the TGFβ2/Smad2/3 signaling pathway. This further supports the hypothesis that in the inflammatory state of chondrocytes, Lumican targets Anxa1 on the chondrocyte cellular membrane, negatively regulating the TGFβ2/Smad2/3 pathway and mitigating the inflammatory state and degeneration of chondrocytes in TMJOA.

**Figure 10:**
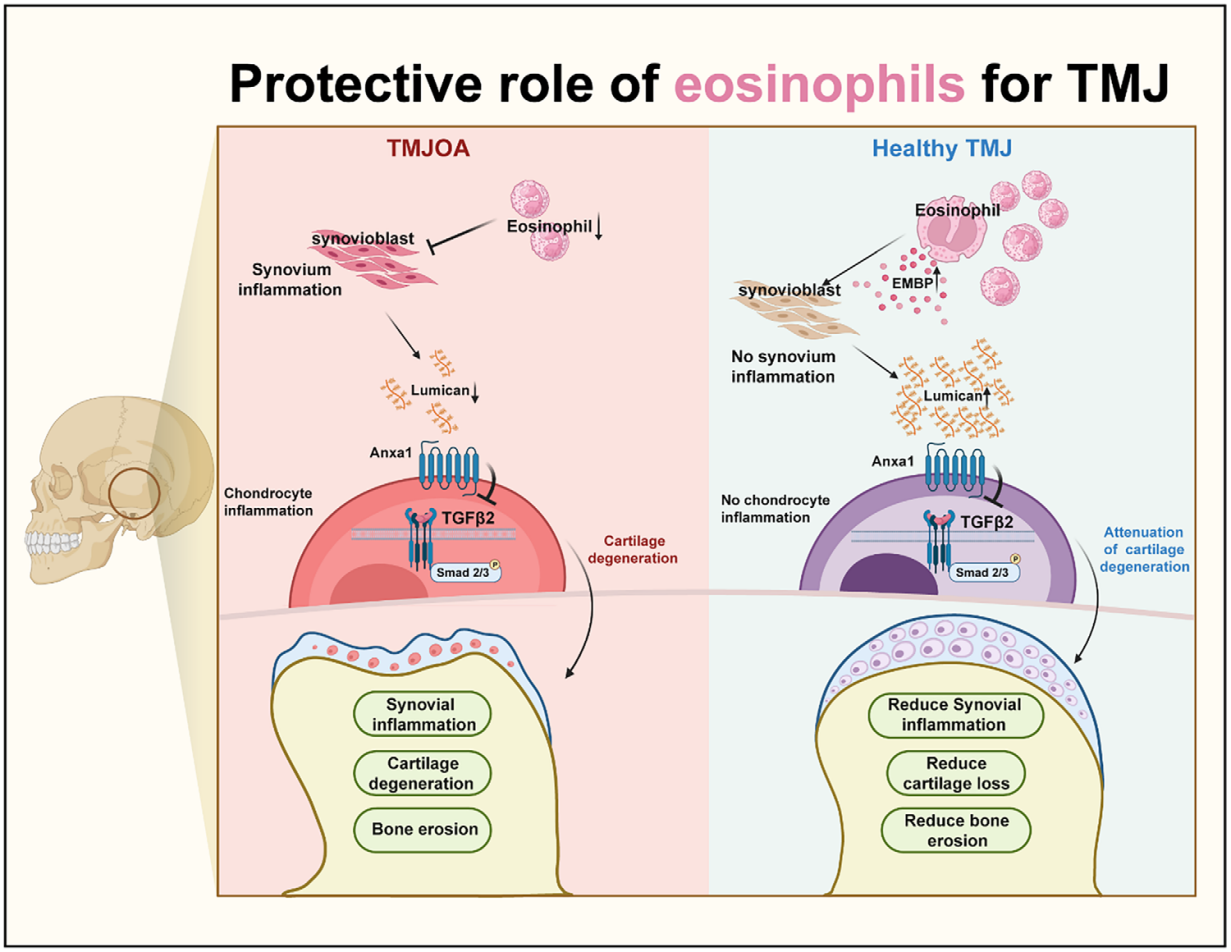
Eosinophils alleviate TMJOA through Lumican mediated TGF‐β signaling pathway to inhibit Smad2/3 phosphorylation via binding Anxa1. EOS in TMJ synovial fluid promotes the secretion of Lumican by synovium. Lumican then binds to Anxa1, inhibiting the phosphorylation of Smad2/3 through the TGF‐β signaling pathway in chondrocytes, thus reducing synovial inflammation, cartilage degeneration, and subchondral bone destruction. Some of the images were generated by biorender.com.

In conclusion, EOS in the TMJ synovial fluid regulates Lumican secretion by synovial tissue, inhibiting TGFβ2‐mediated Smad2/3 phosphorylation of cartilage, thereby reducing synovial inflammation, cartilage degradation, and subchondral bone destruction. This suggests that EOS plays a critical role in alleviating TMJOA progression (**Figure** **10**).

## 3 Discussion

Eosinophils play a protective role in peripheral large joints by modulating inflammatory responses and the phenotype of immune cells, thereby potentially slowing RA progression.^[^ ^24^ ^]^ The pathogenesis of TMJOA and RA is different.^[^ ^1^, ^33^, ^34^ ^]^ In addition, temporomandibular joint differs from peripheral large joints in development, structure, and function.^[^ ^35^, ^36^, ^37^, ^38^, ^39^, ^40^, ^41^, ^42^, ^43^, ^44^, ^61^, ^62^, ^63^, ^64^, ^65^ ^]^ Due to the spatial heterogeneity and pleiotropic functions of eosinophils, their roles may vary significantly across different tissues.^[^ ^66^ ^]^ The distribution,^[^ ^20^, ^21^ ^]^ functions,^[^ ^18^, ^19^ ^]^ and mechanisms of eosinophils in TMJOA remain poorly understood, representing a significant gap in current research. The role of eosinophils in TMJOA remains to be elucidated. Therefore, our study aims to address this void by investigating the role of eosinophils in TMJOA in vivo and in vitro, providing suggestive targeted immunotherapies for TMJOA in the future.^[^ ^67^, ^68^ ^]^ Our study revealed that OVA‐induced allergic asthma enhanced the EOS recruitment in the synovium, alleviating synovial inflammation, cartilage degeneration, and subchondral bone destruction in TMJOA. Furthermore, we introduced TRFK to impede the production of eosinophils. IL‐5 regulates eosinophil differentiation, activation, and survival, making it a key target for depletion.^[^ ^69^, ^70^ ^]^ The IL‐5 neutralizing antibody, TRFK5, effectively reduces eosinophil counts in vitro and in vivo, with consistent validation in murine models.^[^ ^71^, ^72^ ^]^ Its established efficacy and reliability make it the optimal choice for this study. Notably, EOS deficiency exacerbated TMJOA. Mechanistically, EOS promoted Lumican secretion by synovium, and Lumican inhibited the expression of TGFβ2 and reduced Smad2/3 phosphorylation via the TGF‐β signaling pathway of cartilage, mitigating TMJOA.

Traditionally, eosinophils have been considered pathological effector cells that exacerbate inflammatory processes in systemic diseases. However, recent research has highlighted their roles in type II immune regulation.^[^ ^73^ ^]^ Our study observed a higher number and percentage of EOS in healthy subjects' synovial fluid than TMJOA subjects. Although immune cells are scarce in TMJ synovial fluid, the differential distribution of eosinophils in TMJ synovial fluid between healthy and diseased individuals (Figure 1d) is consistent with that observed in the knee joint.^[^ ^23^, ^74^ ^]^ Eosinophils can produce and release granular contents,^[^ ^75^ ^]^ including active substances like galectins, EMBP, and eosinophil cationic protein (ECP).^[^ ^76^, ^77^ ^]^ EMBP, as unique granules expressed by EOS, constitutes the core of eosinophil granules, making up more than 50% of its granular proteins.^[^ ^78^ ^]^ EMBP released when eosinophils degranulate plays a crucial role in inducing bronchial hyperreactivity,^[^ ^79^ ^]^ promotes eosinophil binding to parasites for cytotoxic effects,^[^ ^80^ ^]^ regulates membrane osmotic pressure for antibacterial functions,^[^ ^81^ ^]^ and further activates other immune cells to enhance immune response.^[^ ^82^, ^83^ ^]^ We found a correlation between the Helkimo clinical dysfunction index (Di) and EMBP (Figure 1f). Research on the role of EMBP in joint tissue changes remains scarce. Darja Andreev and colleagues have shown that EPX, another eosinophil protein, helps regulate osteoclast activity and slows osteoarthritis progression.^[^ ^28^ ^]^ Based on those findings and our observations linking Di and EMBP, we hypothesize that eosinophils may also alleviate the progression of TMJOA through EMBP secretion, which requires further experimental validation in the future.

Previous studies have not clarified the specific mechanism by which eosinophils influence TMJOA. Eosinophils impact the activity of osteoblasts and osteoclasts, thereby regulating bone metabolism.^[^ ^84^, ^85^ ^]^ Eosinophils can increase bone thickness and promote the formation of pseudo‐bone tissue, which helps prevent inflammation‐induced bone erosion and supports bone growth.^[^ ^86^ ^]^ This bone‐regulating effect of eosinophils is achieved through their influence on both osteoblast and osteoclast activity, key players in bone metabolism.^[^ ^84^, ^85^ ^]^ The underlying mechanism involves eosinophils secreting osteogenic cytokines such as IL‐4, IL‐5, and IL‐13. These cytokines activate critical signaling pathways, including ILC2‐IL‐9‐Treg and ILC2‐IL‐4/13.^[^ ^86^, ^87^ ^]^ In this way, EOS maintains bone homeostasis and promotes bone repair by modulating the balance of these proteins and cytokines. Future TMJOA therapies could leverage eosinophil‐mediated immune modulation combined with natural adhesives, creating a stable repair environment and supporting cartilage regeneration by mimicking ECM‐like scaffolds.^[^ ^88^ ^]^ Mast cells, derived from a GATA‐1+ progenitor shared with eosinophils,^[^ ^89^ ^]^ release histamine, heparin, and chemotactic factors upon activation,^[^ ^77^, ^90^ ^]^ mediating inflammation and tissue remodeling through degranulation.^[^ ^91^ ^]^ Their altered distribution in peripheral osteoarthritis^[^ ^22^ ^]^ suggests a potential role in joint disease pathogenesis. Future validation using single‐cell sequencing or spatial transcriptomics could further clarify their role in TMJOA.

However, in TMJOA, where maintaining bone homeostasis is crucial, the TGF‐β signaling pathway exhibits a dual regulatory role for bone homeostasis.^[^ ^92^, ^93^, ^94^ ^]^ Studies have shown that distinct branches of the TGF‐β signaling pathway can either support joint homeostasis^[^ ^73^ ^]^ or contribute to pathological remodeling.^[^ ^93^, ^94^ ^]^ For example, dysregulation of the TGF‐β signaling pathway, particularly transcription factors Smad2/3 defects, accelerates cartilage degradation and joint dysfunction,^[^ ^92^ ^]^ while activating the Smad1/5/8 axis promotes chondrocyte hypertrophy and abnormal bone remodeling.^[^ ^95^ ^]^ Given the dual regulatory role of TGF‐β in previous studies, it becomes crucial to investigate further whether eosinophils exert their therapeutic effects in TMJOA via the TGF‐β signaling pathway and how they modulate this pathway.

Eosinophilic granulocytes, derived from the myeloid lineage, are primarily distributed in the synovium.^[^ ^22^ ^]^ Considering this, using RNA sequencing data from synovial tissues of eosinophil‐mediated TMJOA rat models and KEGG enrichment analysis, we identified the involvement of the “Proteoglycans in cancer” and “TGF‐β signaling” pathways (Figure 6d). Further analysis of collagen degradation‐related processes revealed Lumican as a key target within the keratan sulfate proteoglycan component, which negatively regulates TGFβ2 (Figure 7b), suggesting that eosinophil‐mediated treatment may exert its therapeutic effects in TMJOA by modulating this pathway. Lumican's matrix‐stabilizing effects, combined with biomaterials' anti‐inflammatory, sustained drug release properties or immune modulation, offer a promising approach for its future use in targeted TMJOA therapies.^[^ ^88^, ^96^, ^97^ ^]^

The TGF‐β pathway is activated when a ligand binds to TGF‐β receptor I (TβRI), which phosphorylates TGF‐β receptor II (TβRII), leading to the phosphorylation of Smad2/3 proteins,^[^ ^98^, ^99^ ^]^ The Smad2/3 complex then translocate to the nucleus to regulate gene transcription.^[^ ^100^, ^101^ ^]^ It is noteworthy that, in OVA‐treated rats, both RNA and protein levels of TGFβ2, as well as Smad2/3, were obviously downregulated (Figure 6e–g), while EOS depletion by TRFK upregulated TGFβ2 expression, suggesting that OVA‐induced EOS suppressed TGF‐β signaling and its downstream pathways. Protein interaction analyses and literature‐supported reasoning confirmed that Lumican regulates the TGFβ/Smad2/3 pathway (Figure 7a), establishing it as a crucial axis for eosinophil‐mediated protective effects in TMJOA. This observation aligns with previous studies where TGF‐β2 has been shown to exert adverse effects on fibrous and cartilaginous tissues through the Smad2/3 signaling pathway.^[^ ^94^, ^102^, ^103^ ^]^ TGF‐β2 can induce the proliferation and migration of choroidal pericytes via the Smad2/3 pathway,^[^ ^102^, ^104^ ^]^ promoting their transformation into myofibroblasts and exacerbating retinal fibrosis.^[^ ^103^ ^]^ This mechanism helps explain the abnormal synovial hyperplasia and fibrosis observed in the TMJ under the influence of TGF‐β through the Smad2/3 pathway in our study.

Smad protein modifications play a crucial role in regulating Smad2/3 signaling in chondrocytes.^[^ ^105^, ^106^ ^]^ Interestingly, Smad signaling exhibits a dual role in certain scenarios; TGFβ‐induced Smad2/3 phosphorylation promotes chondrogenesis by enhancing Sox9 expression and Col2a1 binding,^[^ ^105^ ^]^ while excessive TGFβ signaling can lead to Smad2/3 phosphorylation that promotes chondrocyte hypertrophy, causing cartilage degeneration. Additionally, when Smad2/3 is phosphorylated without Smad1/5 activation,^[^ ^93^, ^94^ ^]^ it can impair MSC‐mediated chondrogenesis and hinder cartilage repair.^[^ ^107^ ^]^ Therefore, it is plausible that inhibiting Smad2/3 phosphorylation in our study could prevent the adverse effects of prolonged hypertrophy and enhance the chondrogenic potential of progenitor cells to help shift the cartilage balance toward favorable cartilage repair and regeneration.

In TMJOA, a complex crosstalk exists between synovial cells and chondrocytes. The synovium interacts with cartilage through various mechanisms, including ligand‐receptor binding, exosomes, adhesion molecules, and metabolites. Synovial cells establish a relationship with cartilage through the secretion of synovial fluid. Activated synovial tissue exacerbates cartilage inflammation by secreting pro‐inflammatory mediators^[^ ^108^, ^109^ ^]^ and matrix metalloproteinases (MMPs),^[^ ^110^ ^]^ further amplifying the inflammatory cascade in synovial cells.^[^ ^111^, ^112^ ^]^ In turn, increased cartilage wear particles and extracellular matrix fragments produced by cartilage in the synovial fluid interact with SFs via adhesion, phagocytosis, or inflammatory mediator secretion, leading to their differentiation into myofibroblast‐like cells (MFLCs), thereby worsening synovial fibrosis and inflammation^[^ ^110^, ^113^ ^]^ and resulting in a vicious cycle of joint degeneration. Protein‐protein interaction networks (PPI) play a crucial role in analyzing intercellular crosstalk and identifying key functional targets.^[^ ^114^ ^]^ Therefore, using PPI analysis, we constructed a protein interaction network based on overlapping genes between (MIA VS Sham) and (OVA+MIA VS MIA), with potential interactions predicted via the STRING database (Figure 7a). Lumican, a keratan sulfate‐containing leucine‐rich proteoglycan from the SLRP family, regulates cell migration, proliferation, and collagen fiber assembly.^[^ ^58^, ^115^, ^116^ ^]^ Its roles in other tissues, such as inhibiting cell proliferation,^[^ ^117^, ^118^ ^]^ and migration while supporting pre‐osteoblast^[^ ^119^, ^120^ ^]^ survival and differentiation, suggest potential benefits in TMJOA. Additionally, Lumican may act as an endogenous inhibitor of TGF‐β by regulating the pericellular availability of its isoforms, influencing Smad2 activation in the TGF‐β signaling pathway.^[^ ^120^ ^]^ Interestingly, our KEGG pathway analysis indicated that Lumican regulates the TGF‐β signaling pathway through the Keratan Sulfate Proteoglycan pathway (Figure 7b). This also suggested that Lumican could be a key modulator of TGF‐β signaling pathway.

In synovial samples, we validated the role of the Lumican/TGFβ2/Smad2/3 pathway. Unlike previous studies that primarily focused on the role of TGFβ/Smad2/3 in either the synovium^[^ ^121^ ^]^or cartilage^[^ ^93^ ^]^ independently, our research explores how eosinophil‐influenced synovium impacts the TGFβ/Smad2/3 pathway in cartilage. This highlights the TGFβ/Smad2/3 pathway as a key mediator of synovium‐to‐cartilage crosstalk in TMJOA progression. Since Lumican is a secreted protein, it is plausible that synovial‐derived Lumican could act on cartilage. Additionally, the TGF‐β pathway, mediated by Smad2/3, plays a crucial role in the progression of TMJOA.^[^ ^92^ ^]^ Therefore, we hypothesized that the EOS‐mediated increase in Lumican secretion from the synovium might alleviate cartilage degradation through the TGFβ2/Smad2/3 pathway. In vitro, initially, we observed that the direct addition of EOS supernatant or lysate led to changes in Aggrecan levels in rMCCs. However, there was no significant alteration in TGFβ2 levels (Figure 8e; Figure S4f,g, Supporting Information). This suggests that the absence of SFs as a mediating factor may impede the corresponding changes in TGFβ2 in rMCCs. Moreover, in the non‐inflammatory state, rMCCs do not exhibit abnormal expression of TGFβ2.^[^ ^95^ ^]^ When we added EOS supernatant or lysate to SFs and then extracted the conditioned medium, rather than adding it directly to the rMCCs, we noted that the EOS lysate elevated Lumican levels in the SF medium significantly and increased Aggrecan expression in rMCCs, while TGFβ2 levels remained unchanged (Figure 8e‐blue and orange columns; Figure S4f,g, Supporting Information). This indicates that EOS significantly enhances Lumican levels by promoting its secretion from SFs. We hypothesize that this lack of change in TGFβ2 expression of rMCCs may be due to the still non‐inflammatory state of rMCCs and the already high baseline secretion of Lumican by SFs, which may render the additional increase from EOS insufficient to affect TGFβ2 expression in rMCCs.

Furthermore, in the inflammatory state of rMCCs with IL‐1β, we found that Aggrecan expression in rMCCs further decreased, simulating a more severe TMJOA environment. Intriguingly, under these conditions, the addition of EOS supernatant or lysate not only increased Aggrecan expression in rMCCs, but also downregulated TGFβ2 and pSmad2/3 expression in rMCCs (Figure 8e; Figure S4f,g, Supporting Information). Notably, we observed a substantial decline in Lumican levels in the si‐*Lum*‐SFs medium compared to si‐NC‐SFs (Figure 8h). This mirrors the TMJOA state where, in the context of insufficient Lumican secretion from SFs and abnormal activation of TGFβ2 in rMCCs,^[^ ^95^, ^98^, ^99^ ^]^ the introduction of EOS supernatant or lysate promotes increased Lumican release from SFs, thereby reducing TGFβ2 and pSmad2/3 expression of rMCCs and potentially delaying cartilage degradation. Interestingly, our findings indicated that the protective effect of the EOS lysate on rMCCs appeared to surpass that of the EOS supernatant. This result suggests that eosinophils, through their degranulation mechanism, alleviate TMJOA primarily by the targeted release of bioactive substances secreted into the extracellular environment in response to specific stimuli, allowing them to interact with neighboring cells or tissues to initiate or modulate various immune responses,^[^ ^122^, ^123^ ^]^ such as enzymes, cytokines, or granule proteins, such as EMBP we mentioned before (Figure 1e,f).

In contrast, cell lysates represent the entire intracellular content, which may contain inactive or less concentrated forms of these bioactive molecules, resulting in diminished functional effects. The underlying mechanisms warrant further investigation in future studies. Together, these results underscore the OVA/IL‐5‐induced increase in eosinophils promotes elevated Lumican secretion in the synovium, which in turn inhibits Smad2/3 phosphorylation mediated by the TGF‐β signaling pathway of cartilage, alleviating cartilage degradation.

However, we are particularly interested in understanding how Lumican regulates the TGFβ2/Smad2/3 pathway, which represents a deeper exploration of the mechanisms by which Lumican functions in TMJOA. Therefore, using methods such as CO‐IP, LC‐MS/MS, and AlphaFold3 to predict PPI, we discovered that Lumican regulates this pathway by binding to the Anxa1, a membrane protein receptor of chondrocytes under inflammatory conditions, revealing a previously unreported regulatory mechanism of Lumican in the alleviation of cartilage pathology in TMJOA.

Anxa1, a member of the annexin protein superfamily, is a calcium/phospholipid‐binding protein involved in cell proliferation, apoptosis, vesicle transport, differentiation, and inflammatory responses, considered an anti‐inflammatory protein due to its role in immune response regulation.^[^ ^124^, ^125^ ^]^ Our results indicate that Anxa1 plays a protective role in TMJOA. After the knockdown of Anxa1, we observed a downregulation of Aggrecan expression related to collagen matrix synthesis and upregulation of MMP13 and TNF‐α, markers of matrix degradation and inflammation, respectively, in IL‐1β‐induced rMCCs. These findings are consistent with previously reported protective effects of Anxa1 in peripheral joints. In RA synovial fluid, neutrophils rely on Anxa1, with elevated levels of Anxa1 in microvesicles, and induce TGF‐β1 production via its receptor, FPR2/ALX, thereby protecting cartilage.^[^ ^126^ ^]^ Prior bioinformatics analyses also support Anxa1's protective role in OA,^[^ ^127^ ^]^ where nucleus pulposus cells (NPC) seek inflammation relief from neutrophils via the Anxa1‐FPR1 pathway, suggesting Anxa1 as a potential therapeutic target for Intervertebral Disc Degeneration (IDD).^[^ ^128^ ^]^ These findings highlight the cartilage‐protective role of Anxa1 in peripheral joint diseases, such as RA and IDD.

Excitingly, in this study, we discovered that Anxa1 is the key intermediary binding target protein through which Lumican, secreted by the synovium, exerts its inhibitory effect on the TGFβ2/Smad2/3 pathway in cartilage, thereby delaying cartilage degradation, which has not been previously reported.

When screening for potential binding proteins detected by LC‐MS/MS, we first used AlphaFold3 to predict the likelihood of protein‐protein interactions. AlphaFold provides pLDDT EPE, which helps researchers assess the reliability of the predicted structures. It also offers significant advantages in protein‐protein interaction analysis by providing high‐resolution, atomic‐level structure predictions, essential for understanding molecular details. These capabilities make it a powerful tool for studying difficult‐to‐capture protein interactions.^[^ ^129^, ^130^ ^]^

Lumican, characterized by its β‐sheet and helical regions, and Anxa1, which also exhibits helical structures, showed a favorable orientation for potential docking. This structural complementarity increased the likelihood of interaction (Figure 9f). The confidence scores (pLDDT, 50–70) for both proteins in the regions corresponding to the predicted interaction sites and the EPE heatmap exhibiting relatively low uncertainty indicated that the spatial arrangement of these residues may be accurate and well‐placed for a potential binding event.

In the AlphaFold model, the secondary structures of Lumican and Anxa1 were clearly defined, with well‐folded helices and β‐sheets, indicating high confidence in the predicted 3D structures. From the visualization, the binding free energy between Lumican and Anxa1 is ‐69.70 kcal mol^−1^, indicating a strong binding affinity between the two, leading to the formation of a stable complex. Key structural features between Lumican (green) and Anxa1 (purple) illustrated two proteins approached each other in a manner that suggested possible interaction interfaces. The residues involved in potential binding were shown with their side chains (such as Glu, Asp, Lys, Arg), highlighting possible electrostatic and hydrophobic interactions between the proteins (Figure 9g). Specific residue pairs, such as Glu‐20 (Anxa1) and Lys‐69 (Lumican) is likely to form electrostatic‐hydrogen bond interactions, a key mechanism in protein‐protein binding.

Comparing the predicted interaction sites from AlphaFold (Figure 9g) with the peptide sequences identified in the mass spectrometry data (Figure 9h), we find a strong overlap between the regions of Anxa1 predicted to interact with Lumican and the fragments identified in the MS/MS spectrum. The combination of the high‐confidence structural predictions from AlphaFold (with pLDDT scores between 50–70) and the mass spectrometry data increases our confidence in the potential interaction between Lumican and Anxa1.

However, uncertainties remain in the predictions of interaction interfaces and some structures of protein complexes with the AlphaFold model. Therefore, we further validated these findings experimentally. Our results indicate that the knockdown of *Anxa1* reduced the protective effect of Lumican and increased the aberrant expression of the TGFβ2 and phosphorylation of the Smad2/3 pathway in IL‐induced rMCCs (Figure 9k,l). Previous studies have found that in cancers such as basal‐like breast cancer (BLBC) and pancreatic cancer,^[^ ^131^, ^132^ ^]^ Anxa1 expression activates the TGFβ/Smad3 pathway to induce epithelial‐mesenchymal transition (EMT)‐like changes, an activation that can be inhibited by TGFβ type I receptor inhibitors.^[^ ^131^ ^]^ In contrast, the interaction between Anxa1 and FPR2 has been shown to suppress Smad3 phosphorylation in esophageal cancer cells.^[^ ^59^ ^]^ We hypothesize that this differential effect of Anxa1 on TGFβ signaling suggests that Anxa1 may promote Smad3 phosphorylation induced by TGFβ1 while inhibiting Smad3 phosphorylation triggered by TGFβ2, possibly due to differences in the cellular environment.

Regarding whether Anxa1 exerts joint‐protective effects through TGFβ regulation, some studies have indicated that EVs derived from neutrophils of Anxa1‐deficient mice^[^ ^133^ ^]^ can inhibit the expression of cartilage degradation factors induced by IL‐1β stimulation in osteoarthritis without relying on Anxa1. This discrepancy suggests that EVs derived from neutrophils might increase TGFβ1, which could be beneficial in inhibiting chondrocyte hypertrophic differentiation,^[^ ^134^ ^]^ further illustrating the complexity of the TGFβ pathway in cartilage protection. In this study, Lumican secreted by synovium, induced by eosinophils, mediates the inhibition of TGFβ2/Smad2/3 through Anxa1. This suggests that Anxa1, under eosinophil stimulation, also exerts an effect on reducing TGFβ2 signaling. Additionally, previous literature linking Anxa1 with increased Th2 cell infiltration^[^ ^132^ ^]^ provides further evidence that Anxa1 may play a role in the alleviation of TMJOA induced by eosinophils. Our findings highlight the mechanistic role of Lumican in modulating TGFβ signaling via its binding to Anxa1, which is critical for the cellular crosstalk between synovium and cartilage in TMJOA.

In conclusion, our results demonstrate that eosinophils represent a crucial, previously underappreciated connection between the innate immune system and the alleviation of TMJOA. Lumican exerts its effect by binding to the Anxa1 receptor on the surface of chondrocytes, thereby inhibiting the TGFβ2/Smad2/3 axis, which plays a key role in slowing cartilage degradation. Targeting the Lumican/TGFβ2/Smad2/3 axis, with a focus on Anxa1 as a key mediator in Lumican's inhibition of this pathway, may provide therapeutic strategies for TMJOA remission, highlighting their significant role in slowing disease progression.

## 4 Experimental Section

### Human Subjects

It was enrolled 28 subjects with TMJOA (4 male, 24 female, mean age: men, 35.0 ± 7.7 years, women, 27.8 ± 3.3 years) and 19 healthy subjects (7 male, 12 female, mean age: men, 28.1 ± 0.7 years, women, 27.5 ± 1.1 years) in a single‐center clinical study at the Stomatological Hospital affiliated with Fujian Medical University. The study was approved by the Ethics Review Committee (Approval No. 2 024 088). The research followed the Research Diagnostic Criteria for Temporomandibular Disorders (RDC/TMD) IIIb guidelines, with inclusion criteria based on the clinical and radiographic diagnosis. This study adhered to the Comprehensive Clinical Trial Reporting Standards (CONSORT) guidelines for reporting, focusing on the clinical and radiographic diagnosis of TMJOA in patients aged 18 and older. Exclusion criteria included uncontrolled systemic disease, neurological disorders, previous temporal‐mandibular joint surgery, and malignant disease in the head and neck region. Data collected included temporomandibular joint synovial fluid analysis, CBCT imaging, visual analog scale (VAS) pain scores, and the Helkimo Clinical Dysfunction Index.

### Animal Models

The animal experimental protocol was approved by the Ethics Committee of Fujian Medical University (Approval No. IACUC‐FJMU‐2023‐0204). Six‐week‐old male Sprague‐Dawley (SD) rats were obtained from the Animal Experiment Center at Fujian Medical University and randomly assigned to different groups. The rats were housed under a 12 h light/dark cycle with unrestricted access to food and water. To establish the ovalbumin‐induced hyper‐eosinophilia rat model, 1 ml of PBS containing 10 mg of ovalbumin and 20 mg of aluminum hydroxide (Al (OH)_3_) was administered via intraperitoneal injection for sensitization. On day 15, the rats were exposed to a 2% ovalbumin aerosol in an induction chamber once daily for 7 days to induce asthma. The TMJOA model in this study was established using sodium iodoacetate (MIA) via intra‐articular injection into the superior joint cavity of the temporomandibular joint (TMJ), a procedure that mimics clinical practice.^[^ ^54^, ^135^, ^136^ ^]^ Rats were positioned with their mandibular incisors gently pressed down to achieve passive maximum mouth opening. The injection site was located ≈5 mm anterior to the external ear and above the zygomatic arch, confirmed by palpating the condyle and glenoid fossa at the most depressed point of the preauricular skin. A needle was inserted anterosuperior‐medially to a depth of 2–5 mm, where bony resistance was felt. After confirming no blood aspiration, 50 µL of 1 mg sodium iodoacetate solution was injected slowly into the TMJ cavity, followed by a gentle massage to ensure proper distribution. Injection of MIA leads to joint swelling, cartilage degradation, and synovial inflammation, closely mimicking the symptoms of TMJOA and making it a widely used model for research.^[^ ^137^, ^138^ ^]^ In the eosinophil‐depleted model, the anti‐IL5 neutralizing antibody TRFK5 (Invitrogen, 14‐7052‐85) was administered at a dose of 17.5 µg per rat, three times a week, with an equal volume of isotype control antibody injected into the control group. Two weeks after TMJOA induction, the rats were euthanized by an overdose of intraperitoneally administered pentobarbital sodium. TMJ and surrounding tissues were harvested for histological and flow cytometry analysis.

### Flow Cytometry Analysis

The left lung lobe was excised for lung tissue processing, and fat, fibrous, and necrotic tissues were carefully removed. The tissue samples were washed with PBS, minced, centrifuged, and collected. The lung tissue was then dissociated using the Lung Tissue Dissociation Kit (Bioleader, BL‐TDK‐F), and the filtrate was obtained through filtration. Red blood cells were lysed using Red Cell Lysis Buffer (Solarbio, R1010) at 4 °C, resuspension, and cell counting. The synovial tissue of the temporomandibular joint was excised, minced, filtered, and digested with Type II collagenase while shaking. After digestion, the tissue was resuspended and counted. To stain the cells, Fixable Viability Stain (BD, 564 997) was applied to 700 µL of cells, followed by washing and adding FcR blockers (BD, 550 270). Surface marker staining for eosinophils was performed using APC‐Cy7 Mouse Anti‐Rat CD45 (OX‐1) (BD, 561 586) and V450 Mouse Anti‐Rat CD11B (WT.5) (BD, 562 108). For intracellular eosinophil marker staining, the cells were fixed and permeabilized using the Fixation/Permeabilization Kit (BD, 554 714), followed by staining with EMBP (F‐6) FITC (Santa Cruz, sc‐365701‐FITC). After staining and washing, the cells were resuspended for flow cytometry analysis. The AF488 channel was used for FVS 700, the APC‐Cy7 channel for CD45, and the BV421 channel for CD11B. Staining and analysis were carried out according to the manufacturer's protocol.

### Immunohistochemical Staining

Paraffin‐embedded tissue sections were used for immunohistochemistry (IHC) and immunofluorescence (IF) staining to assess the expression of Lumican and TGFβ2 in the temporomandibular joint. After deparaffinization and rehydration, a pepsin repair solution (MXB, DIG‐3009) was applied for epitope retrieval, following the manufacturer's instructions. The UltraSensitive SP (rabbit) IHC Kit (MXB, Kit‐9707) was used for staining. Endogenous peroxidase activity was blocked using 3% hydrogen peroxide, and non‐specific signals were prevented by blocking with 5% goat serum. The slides were then incubated overnight at 4 °C with rabbit anti‐mouse Aggrecan and MMP‐13 antibodies. After washing, the slides were treated with biotin‐labeled goat anti‐rabbit IgG and stained with DAB. Hematoxylin was used for counterstaining to visualize the nuclei, and the slides were mounted for analysis.

### Immunofluorescence

Paraffin‐embedded tissue sections were used for immunohistochemistry (IHC) and immunofluorescence (IF) staining to assess the expression of Aggrecan and MMP13 in the temporomandibular joint. After deparaffinization and rehydration, a pepsin repair solution (MB, DIG‐3009) was applied for epitope retrieval. The sections were blocked with 5% goat serum at room temperature for 1 h. The tissue was then incubated overnight at 4 °C with aggrecan antibody (Wanlei, WL02316, 1:200) and MMP13 antibody (Proteintech, 1:200). Following incubation, Alexa Fluor 488‐conjugated secondary antibodies were applied, and DAPI (Beyotime) was used to counterstain the nuclei. The samples were sealed and observed using confocal microscopy (Olympus 3000) for imaging and analysis.

### Enzyme‐Linked Immunosorbent assay (ELISA)

Supernatants of SF were also collected, and the concentration of Lumican (BOSTER, EK1262) was determined using an ELISA kit.

### Isolation and Ex Vivo Culturing of Rat bmEOS

In vitro cultures were conducted as described in previous studies. Briefly, bone marrow cells were collected from the femur of rats, filtered, and red blood cells were lysed. The cells were then cultured in RPMI 1640 medium supplemented with 10% fetal bovine serum, 100 IU mL^−1^ penicillin/streptomycin, 2 mM glutamine, 25 mM Hepes, 1x non‐essential amino acids, 1 mM sodium pyruvate (Gibco), and 50 µM β‐mercaptoethanol. The cells were seeded at a concentration of 10^6^ cells mL^−1^ in RPMI 1640 and supplemented with 100 ng mL^−1^ stem cell factor (SCF, Rat, HEK293) and 100 ng mL^−1^ FLT3 ligand (PeproTech). On day 4, the medium was replaced with fresh medium containing 20 ng mL^−1^ recombinant rat IL‐5 (MCE, HY‐S‐240111S686), and the medium was changed every two days for 8 days. On day 12, the cells were harvested for flow cytometry analysis, and cell smears were prepared for identification using Giemsa staining under a microscope.

### RNA Sequencing

Rat Synovial tissues were collected and sent to Wuhan Kangsi Technology Co., Ltd. Total RNA was extracted from the TMJ synovial tissue using TRIzol Reagent (Invitrogen, Cat. No. 15 596 026)^[^ ^104^ ^]^ Following RNA extraction, DNA was digested using DNase I. The RNA quality was assessed by measuring the A260/A280 ratio with a Nanodrop OneC spectrophotometer (Thermo Fisher Scientific). Gene exon reads were counted using featureCounts (Subread‐1.5.1; Bioconductor), and RPKM values were calculated. Differential gene expression between groups was determined using the edgeR package (version 3.12.1), with statistical significance defined by a p‐value cutoff of 0.05 and a fold‐change threshold of 0.5. Gene ontology (GO) analysis and Kyoto Encyclopedia of Genes and Genomes (KEGG) pathway enrichment for differentially expressed genes was conducted using KOBAS software (version 2.1.1), with a significance threshold of *P* < 0.05.

### Protein‐Protein Interaction Analysis

Overlapping genes with more than 30 RPKM between (Combination VS MIA) and (MIA VS Sham) were selected to analyze protein‐protein interaction based on known interactions or structural characteristics using STRING 12.0. A protein interaction network was built by mapping the validated interactions. Analyze the biological significance of PPIs by studying the KEGG enrichment pathways.

### Quantitative Real‐Time Reverse Transcription Polymerase Chain Reaction (qRT‐PCR)

The rat temporomandibular joint cartilage and synovium were cleaned with PBS, and Trizol reagent was added. The tissue was mashed in liquid nitrogen, and total RNA was extracted using chloroform, isopropyl alcohol, and ethanol, following the protocol. cDNA was synthesized from 1000 ng of total RNA using the PrimeScript RT kit with gDNA Eraser (Takara, Japan). TB Green Premix Ex Taq II (Takara, Japan) was used in a real‐time PCR system (QuantStudio5, Thermo Scientific, USA) to evaluate the mRNA levels of *Smad3*, *Smad2*, *Tgfβ2*, *Lumican*, *Bgn*, *Serpinh1*, *Il‐10, Col2a1, Aggrecan*, *Mmp13, Mmp3, Adamts5, Cox‐2* and *Tnf‐α*. *Glyceraldehyde 3‐phosphate dehydrogenase* (*Gapdh*) was used as the internal reference. The data were analyzed using the 2^‐ΔΔCt method. All primer sequences used in this study were listed (Table S3, Supporting Information).

### Western Blot

For animal tissue samples, rat TMJ cartilage and synovium were washed with PBS, and the tissue was ground in liquid nitrogen using a pestle. For cell samples, cells were washed twice with PBS and then lysed on ice using RIPA lysis buffer (Beyotime, P0013K, China) containing protease and phosphatase inhibitors (Abmole, *M7528*, USA) for 15 min. The lysate was sonicated and centrifuged at 15 000 × g for 5 min at 4 °C, and the supernatant was collected. Protein concentrations were measured using the BCA protein assay kit (Beyotime, China). Protein samples were mixed with 5x loading buffer (Beyotime, P0015L, China) and heated at 100 °C for 10 min. Proteins were separated using 4%–20% SDS‐PAGE (ACE, ET15420Gel, China) and transferred onto a 0.22 µm polyvinylidene fluoride (PVDF) membrane (Millipore, GVWP04700, USA). Membranes were blocked with 5% BSA for 3 h at room temperature and then incubated overnight at 4 °C with primary antibodies. After washing, the membranes were incubated with HRP‐conjugated secondary antibodies for 1 h at room temperature. Protein bands were detected using a chemiluminescence detection kit (Biosharp, BL523A, China). The primary antibodies used included Aggrecan (WanleiBio, WL02316, 1:1000), Col2α1 (Servicebio, GB11021, China, 1:1000), MMP13 (Proteintech, 18165‐1‐AP, 1:1000), MMP3 (CST, 14351S, 1:1000), TNF‐α (Zenbio, 346 654, 1:1000), and IL‐10 (Zenbio, 502 171, 1:1000).

IAn indirect co‐culture system was used for grouping: 1) To test the direct effect of Lumican on rMCCs, SFs were stimulated with 5 ng mL^−1^ IL‐1β for 12 h to induce inflammation, and SFs supernatant was extracted as a conditioned medium to culture rMCCs. Different concentrations of Lumican (1.7 mM, 17 mM) were added to the rMCCs medium. rMCCs proteins were extracted after 12 h of culture for protein Western Blot analysis. 2) To test the effect of Lumican secretion by SFs on rMCCs, 5 ng mL^−1^ IL‐1β was used to stimulate SFs with conditional knockdown of Lumican for 12 h to induce inflammation. The supernatant of SFs was extracted as a conditioned medium to culture rMCCs. 3) To test the effect of Lumican secreted by SFs on rMCCs, 5 ng mL^−1^ IL‐1β was used to stimulate SFs with conditional knockdown of Lumican for 12 h to induce inflammation. The supernatant of SFs was extracted as a conditioned medium to culture rMCCs, and Lumican was added to the rMCCs medium. rMCCs proteins were extracted after 12 h of culture for protein immunoblot analysis. 4) In order to test whether EOS played a role in rMCCs by stimulating SFs to secrete Lumican, EOS were isolated from rats and cultured. The supernatant of EOS was extracted, or the lysate was obtained by repeated freeze‐thaw and added into SFs as a conditioned medium. The lysates and supernatants of EOS were harvested and added respectively to SFs with the original medium in either unmixed or 1:1 ratio and treated for 12 h. Their supernatant was used to treat IL‐1β‐induced rMCCs.

### Micro‐Computed Tomography (µCT) Analysis

The collected condylar specimens were scanned by micro‐CT instrument (NEMO Micro CT, NMC‐200, PINGSENG Healthcare (Kunshan) Inc), and analyzed using the software (Avatar3, PINGSENG Healthcare (Kunshan) Inc), which included 3D reconstruction and cross‐sectional evaluation. The scanning parameters were set at 70 kV voltage, 200 µA current, and high resolution. Images were processed using Cruiser software and reconstructed with the Avatar system to generate 3D models of the trabecular bone. Three cubic regions of interest (each 0.5 × 0.5 × 0.5 mm^3^) were selected 0.5 mm below the condylar subchondral bone. In these regions, key parameters were calculated, including bone volume fraction (BV/TV), trabecular separation (Tb.Sp, mm), trabecular thickness (Tb.Th, mm), bone mineral density (BMD), and structural model index (SMI), tissue mineral content (Tb.TMC, mg), bone surface to volume ratio (Tb.BS/TV, 1/mm), and degree of anisotropy (DA).

### Histomorphometry of Bone Deposits

Calcein and alizarin red markers were used to evaluate the rats' subchondral bone mineral deposits and bone formation. Calcein sodium salt (Solarbio, C7601, 30 mg kg^−1^) was injected 9 days before sacrifice, and alizarin red (40 mg kg^−1^) was injected 2 days before sacrifice. After euthanasia, the condylar subchondral bone was collected and fixed in 4% paraformaldehyde (Biosharp, BL539A) at 4 °C for 24 h with gentle stirring. The bone samples were immersed in 15% and 30% sucrose solutions for 24 h each. Once permeated with sucrose, the samples were embedded in the OCT compound and frozen at −80 °C. Sections were cut using a Leica freezing microtome (Leica, CM1950), with 30 µm frozen sections prepared using CryoJane adhesive tape, following previously reported methods. Norland Optical Adhesive 63 (Norland Products, 6301, Cranbury, NJ, USA) was applied to the slides, and UV curing (Kylin‐Bell, GL‐3120) was used to seal the sections. Confocal laser scanning microscopy was used to observe the fluorescent labeling of calcein (excitation 488 nm/emission 500–550 nm) and alizarin red (excitation 543 nm/emission 580–670 nm).

### RNA Knockdown

*Lumican* expression was transiently inhibited using small interfering RNA (siRNA) produced by Shanghai Genepharma Company. The siRNA was transfected into cells using Lipofectamine 3000 transfection reagent (Invitrogen, L3000001). The siRNA sequence used was as follows: forward (5′‐3′): CUGGCAUCAAGUACCUUUATT, and reverse (5′‐3′): UAAAGGUACUUGAUGCCAGTT. *Anxa1* expression was also transiently inhibited using siRNA, and was transfected as mentioned before. The siRNA sequence of *Anxa1* used was as follows: forward (5′‐3′): GAAGGGACUUGGAACAGAUTT, and reverse (5′‐3′): AUCUGUUCCAAGUCCCUUCTT.

### Membrane Protein Extraction

Membrane proteins were extracted using a Membrane and Cytosol Protein Extraction Kit (Beyotime, P0033). Chondrocytes treated with 10 ng mL^−1^ IL‐1β for 12 h were scraped using a cell scraper and centrifuged at 600 g for 5 min to pellet the cells. The supernatant was discarded, and the pellet was centrifuged again at 600 g for 1 min to remove residual liquid on the tube wall and further concentrate the cells. Subsequently, 1 mL of Membrane Protein Extraction Reagent A (supplemented with PMSF) was added to the cell pellet. The cells were incubated on ice for 10–15 min, followed by two freeze‐thaw cycles alternating between liquid nitrogen and room temperature to disrupt the cells. The lysate was centrifuged at 700 g at 4 °C for 10 min, and the supernatant was carefully transferred to a new centrifuge tube. The supernatant was then centrifuged at 14 000 g at 4 °C for 30 min to pellet cell membrane fragments. The supernatant was discarded, and the pellet was resuspended and centrifuged again at 14 000 g at 4 °C for 5 min. The resulting supernatant was collected as the membrane protein solution, which was subsequently dissolved in NP‐40 for further Co‐IP experiments (BOSTER, AR0107).

### Co‐Immunoprecipitation (Co‐IP)

Protein A/G Magnetic Beads (MCE, USA) were resuspended thoroughly, and 25–50 µL of beads were transferred to a 1.5 mL EP tube. The beads were washed three times with Wash Buffer (BOSTER, AR0107) using a magnetic rack to separate the beads from the supernatant. His‐tagged Mouse McAb (Proteintech, 66005‐1) and His‐tagged recombinant Lumican protein (CUSABIO, CSB‐EP013234RA) were added to the membrane protein sample and incubated with gentle mixing on a rotator at 4 °C for 2 h respectively, serving as the antigen sample. Separately, 400 µL of diluted His antibody (Proteintech, 66005‐1) was added to the prepared magnetic beads, resuspended thoroughly, and incubated on a rotator at 4 °C for 2 h. The beads were then magnetically separated, and the supernatant was discarded. After four rounds of washing with Wash Buffer, 400 µL of the prepared antigen sample was added to the beads, resuspended thoroughly, and incubated on a rotator (room temperature for 30 min, then overnight at 4 °C). The beads were magnetically separated, and the supernatant was discarded. After washing, the beads were collected, lysed, and denatured for subsequent Western blot analysis.

### Protein Silver Staining

Silver staining was performed using a Rapid Silver Stain Kit (Beyotime, P0017S). After completing electrophoresis for Western blotting, the gel was fixed for 40 min, washed with 30% ethanol, and rinsed with water for 10 min. The gel was then sensitized for 2 min, followed by two 1 min washes with water. Silver staining was performed for 10 min, followed by rinsing with water and development. The reaction was stopped at the appropriate stage to visualize the protein bands.

### Liquid Chromatography‐Tandem Mass Spectrometry (LC‐MS/MS)

After performing the CO‐IP experiment and denaturing the protein samples, Western Blot was conducted. Following electrophoresis and silver staining, the bands corresponding to IgG and the target protein Lumican were excised and sent to Shanghai Luming Biotechnology Co., Ltd for LC‐MS/MS‐based proteomic analysis. The gel bands underwent destaining, trypsin digestion, and subsequent mass spectrometric analysis. Data acquisition was followed by database searching, with the search parameters set as MS1 tolerance of 10 ppm and MS2 tolerance of 0.02 Da. Proteins were analyzed qualitatively and quantitatively.

### Prediction of Protein‐Protein Interactions Using the AlphaFold3 Model

Amino acid sequences of the proteins were retrieved from UniProt (https://www.uniprot.org/). These sequences were subsequently submitted to the online AlphaFold 3 prediction model platform for analysis. Five potential interaction models were generated, and Model 0 was selected for further analysis based on its scoring. The predicted protein interaction sites were identified and visualized using PyMOL, highlighting the specific interacting amino acids.

### Statistical Analysis

All statistical analyses were conducted using SPSS version 26.0 (SPSS Inc., Chicago, IL, USA) and Graph‐Pad Prism software 9.3.0. The data were preprocessed to exclude extreme values. Data are presented as mean ± standard error of the mean (SEM). Data normality was examined by the D'Agostino‐Pearson test. One‐way ANOVA Dunnett's test was applied (normally distributed data) while the Mann‐Whitney test was used (non‐normally distributed data) for comparisons between multiple groups. The unpaired T‐test was used (normally distributed data), while the Kruskal‐Wallis test was used (non‐normally distributed data) for comparisons between the two groups. Correlations between parameters for human sample analysis were assessed using the Pearson correlation coefficients analysis as appropriate. Statistical details (e.g., sample size (n) of animals/participants per group, probability (*P*) value, data presentation, and the meaning of the significance symbol, etc.) for each experiment can be found in the figure legends. *P* value of less than 0.05 was considered statistically significant, with significance levels indicated as *P < 0.05; **P < 0.01; ***P < 0.001.

## Conflict of Interest

The authors declare no conflict of interest.

## Supporting information



Supporting Information

## Data Availability

Research data are not shared.
