# Supplementary material for: Eosinophils‐Induced Lumican Secretion by Synovial Fibroblasts Alleviates Cartilage Degradation via the TGF‐β Pathway Mediated by Anxa1 Binding
Source: Adv Sci (Weinh). 2025 Mar 24;12(29):2416030. doi: 10.1002/advs.202416030 (PMC12362735; doi:10.1002/advs.202416030)
Supplement: Supplementary file 1 — Supporting Information [file ADVS-12-2416030-s001.docx]

**Eosinophils-Induced Lumican Secretion by Synovial Fibroblasts Alleviates Cartilage Degradation Via the TGF-β Pathway Mediated by Anxa1 Binding** **Author:** Wenqian Chen^a, b, 1^, Yuwei Zhou^a, b, 1^, Wenxiu Yuan^a, c^, Yanjing Ou^a, b^, Hanyu Lin^a, b^, Kaixun He^a, b^, Xueshen Qian^a^, Huachen Chen^a, b^, Chengchaozi Wang^a, b^, Jie Lu^a, b^, Weiping Chen^a, b^, Dexiong Li^a, b^, Jiang Chen^a, b,^ *

**Supplementary Figures and Tables**


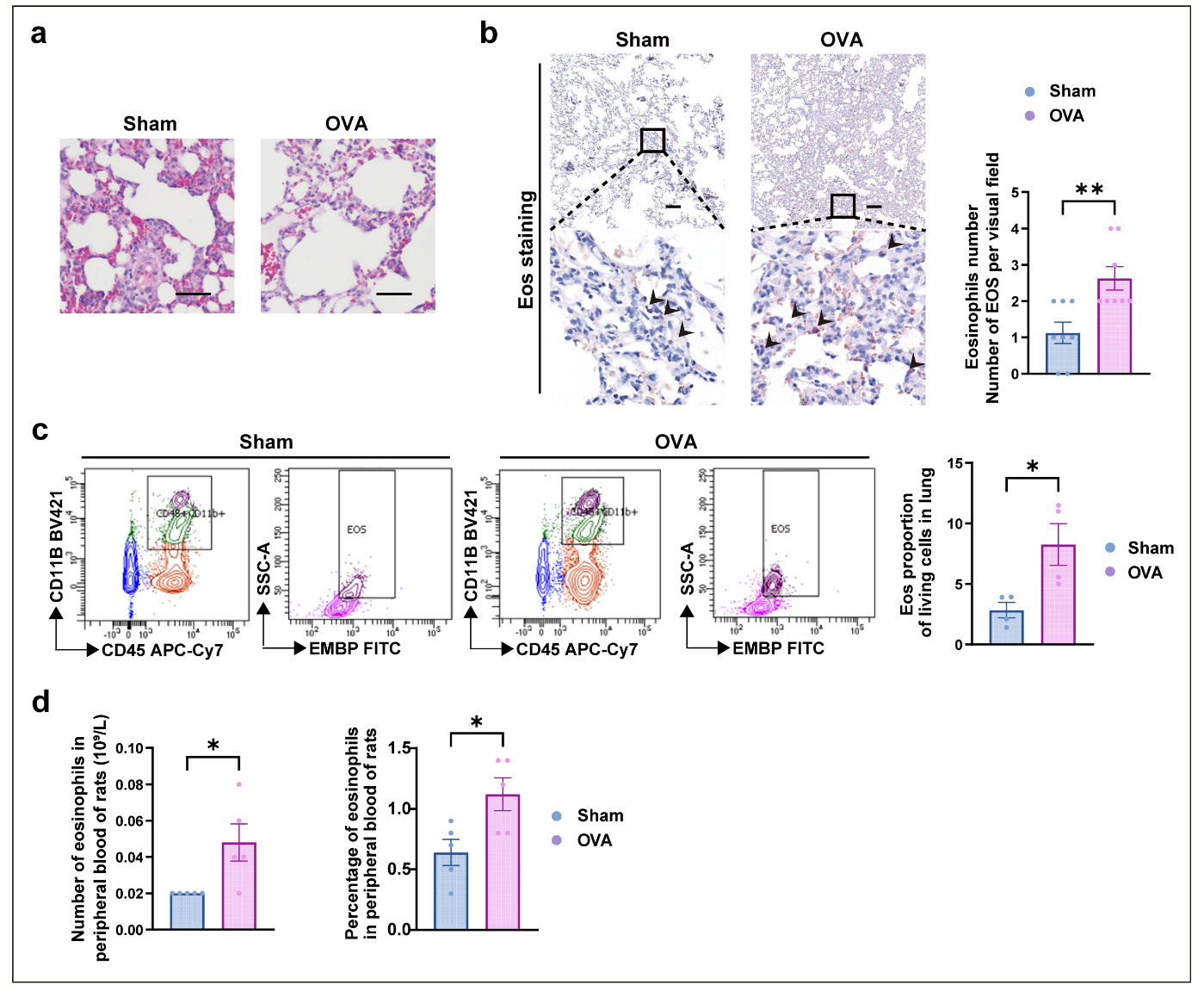


**Figure. S1| Ovalbumin (OVA)-induced hyper-eosinophilia asthma was constructed.** **a** H&E staining of rat lung tissue. Scale bar, 100 μm. **b** EOS staining of rat lung tissue and EOS per visual field were measured. The black arrows indicated EOS. Scale bar, 100 μm. **c** Quantification of EOS (CD11b^+^CD45^+^EMBP^+^cells) in the lung of rats with OVA treatment compared with Sham group analyzed by flow cytometry. **d** The number and proportion of eosinophils in the peripheral blood of rats. Statistical test: Unpaired T-test. **P*<0.05, ***P*<0.01.


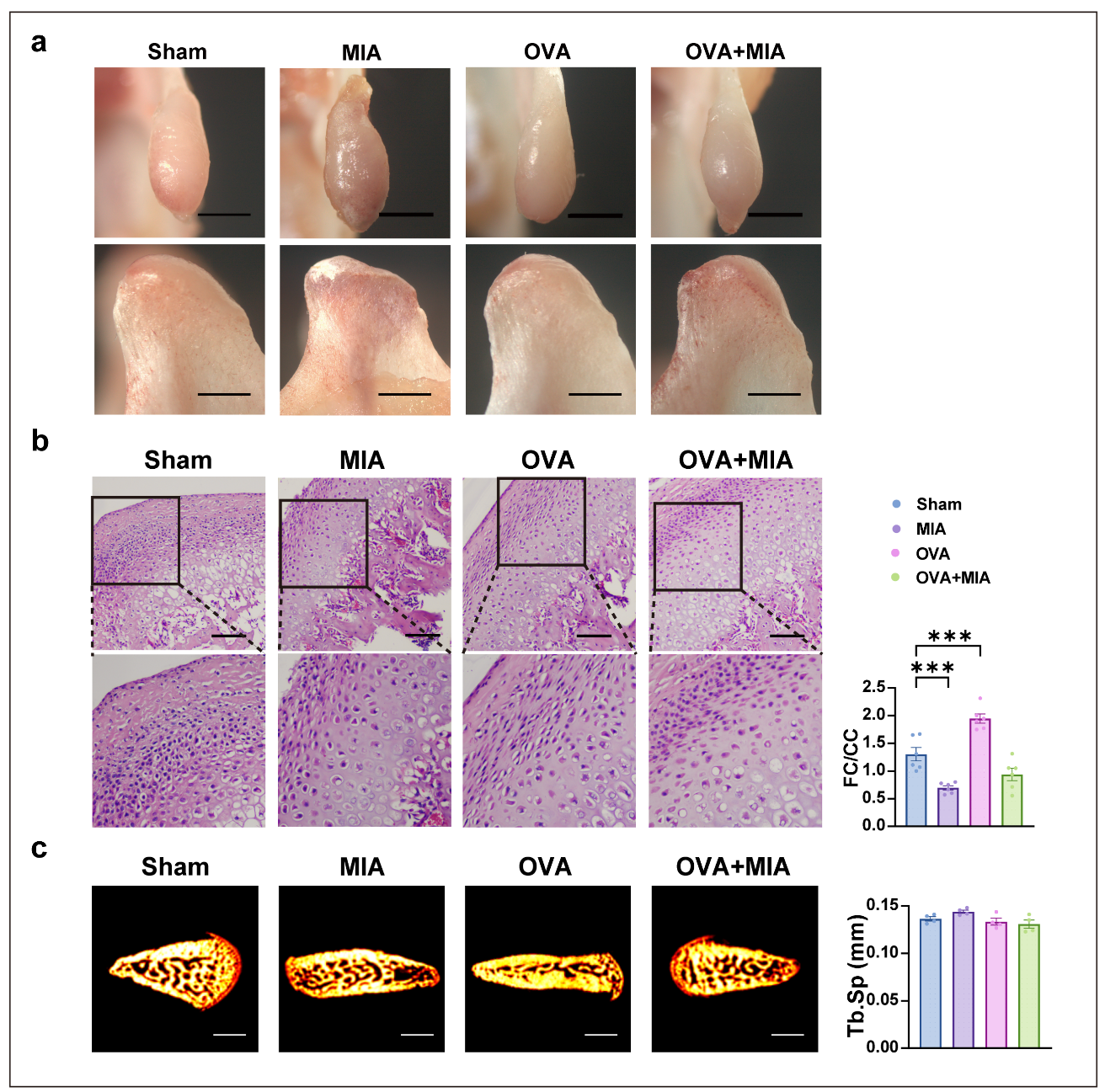


**Figure. S2| OVA-induced hyper-eosinophilia asthma causes regression of synovial, cartilage, and subchondral bone deterioration of TMJOA. a** Horizontal and sagittal view of the condyles in rats. Scale bar, 2 mm. **b** H&E staining and FC/CC analysis of rat cartilage. Scale bar, 100 μm. **c** Micro-computed tomography and analysis of trabecular separation (Tb.Sp, mm) related to bone deterioration. Scale bar, 1 mm. Statistical test: One-way ANOVA Dunnett’s test. ****P*<0.001.


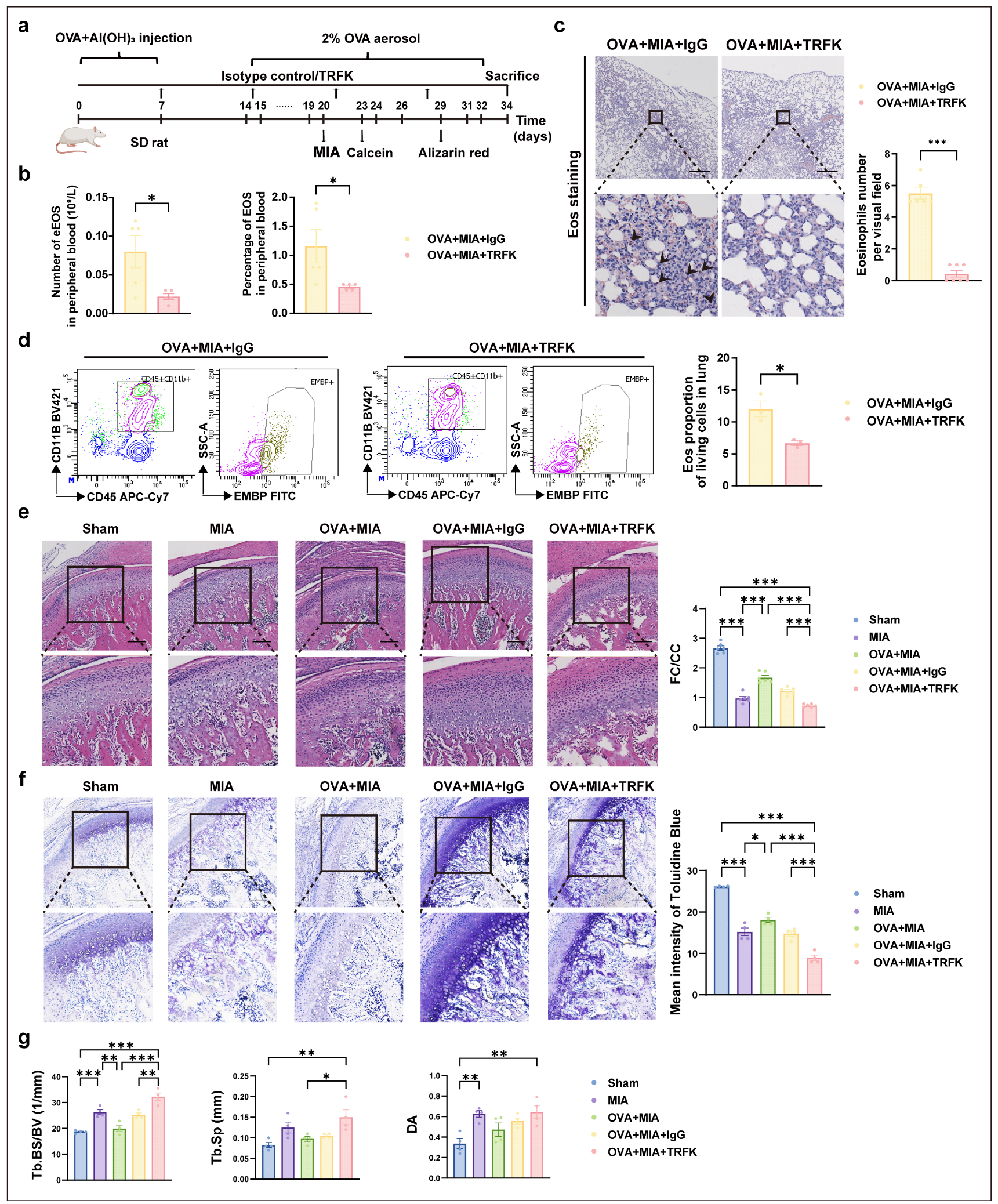


**Figure. S3| Eosinophils are the main effector cells driving asthma-induced resolution of TMJOA by OVA.** **a** An experimental outline of OVA-induced allergic hyper-eosinophilia asthma and MIA-induced temporomandibular osteoarthritis in wild-type (IgG) or EOS-deficient (TRFK) male rats. **b** The number and proportion of eosinophils in the peripheral blood of rats. **c** EOS staining of rat lungs and EOS per visual field were measured. The black arrows indicated EOS. Scale bar, 250 μm. **d** Quantification of EOS (CD11b^+^CD45^+^EMBP^+^cells) in the lung of rats with TRFK treatment compared with IgG group analyzed by flow cytometry. **e** H&E staining and FC/CC analysis of rat cartilage Scale bar, 100 μm. **f** Toluidine blue-stained cartilage of rats. The intensity of toluidine blue per cubic millimeter was analyzed. Scale bar,100 μm. **g** Statistical analysis of BV/TV, Tb.Th, TB.TMC and SMI of rats by micro-computed tomography. Statistical test: b-d, Unpaired T-test. e-g, One-way ANOVA Dunnett’s test. **P*<0.05, ***P*<0.01, ****P*<0.001.

**
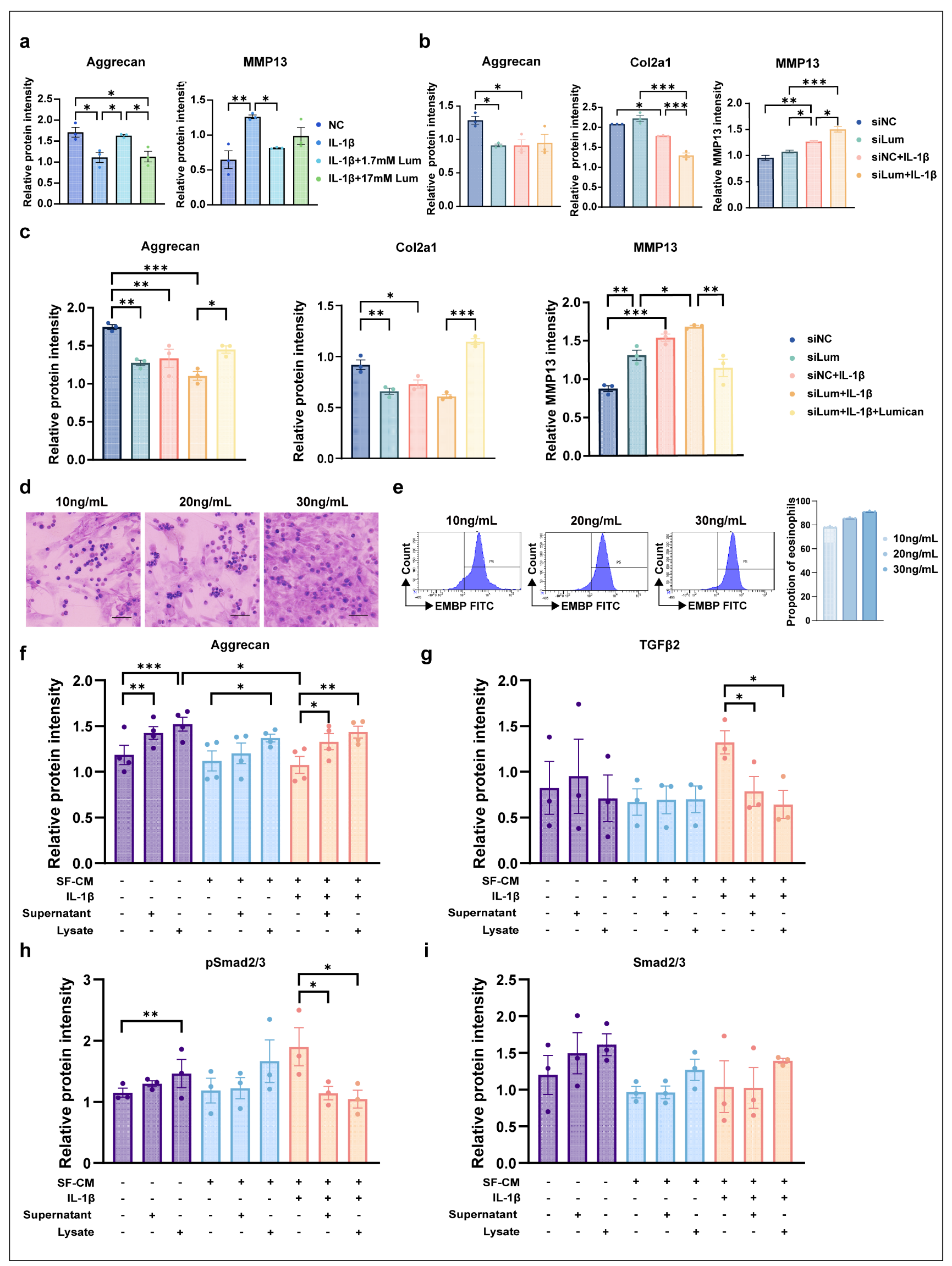
**

**Figure. S4| EOS promotes the secretion of Lumican by synovial fibroblasts and reduces chondrocyte degradation in vitro. a** Western Blotting analysis of Aggrecan and MMP13 for rMCCs in **Figure. 8a** with SFs supernatants cultured for 12 hours **b** Western Blotting analysis of Aggrecan, Col2a1, and MMP13 for rMCCs in **Figure. 8b** with SFs supernatants cultured for 12 hours. **c** Western Blotting analysis of Col2a1, Aggrecan, and MMP13 for rMCCs in **Figure. 8c** with SFs supernatants cultured for 12 hours. **d** Representative cell centrifuge smear preparation from rat eosinophils stimulated with different concentrations of IL-5 recombinant protein at day 12 stained with a modified Giemsa protocol. Scale bar, 50 μm. **e** Flow cytometry analysis of rat eosinophils cultured in vitro stimulated with different concentrations of IL-5 recombinant protein. **f-i** Western Blotting analysis of Aggrecan, TGFβ2，pSmad2/3, Smad2/3 for rMCCs in **Figure.8e**. Statistical test: One-way ANOVA Dunnett’s test. **P*<0.05, ***P*<0.01, ****P*<0.001.

**Supplementary Tables**

**Table S1 | Mann-Whitney statistical analysis of temporomandibular joint visual analog scale (VAS) score in the control and diseased groups. Median (P25, P75)**

|  | Number | VAS score | Z | *p* |  |
| --- | --- | --- | --- | --- | --- |
| Control | 28 | 0(0~2) | -2.704 | 0.007 |  |
| TMJOA | 19 | 4(0~6) |  |  |  |

Note: The graphs show the median VAS values in healthy and diseased individuals, with 25% and 75% percentiles in parentheses.

**Table S2 | Mann-Whitney statistical analysis of Helkimo Clinical Dysfunction Index (Di) in the control and diseased groups. Median (P25, P75)**

|  | Number | Helkimo score | Z | *p* |  |
| --- | --- | --- | --- | --- | --- |
| Control | 28 | 1(0.5~2) | -4.373 | 0.000 |  |
| TMJOA | 19 | 5(4~8) |  |  |  |

Note: The chart shows the median Helkimo Clinical Dysfunction Index in healthy and diseased individuals with 25% and 75% percentiles in parentheses.

**Table S3** **| Rat qPCR primers forward and reverse**

| **Gene** | **Forward** | **Reverse** |
| --- | --- | --- |
| *Gapdh* | 5’ ACGGCAAGTTCAACGGCACAG3’ | 5’CGACATACTCAGCACCAGCATCAC3’ |
| *Smad3* | 5’TCGTCCATCCTGCCCTTCACC3’ | 5’CACCAAGCTCTTGACCGCCTTC3’ |
| *Smad2* | 5’ATGTCGTCCATCTTGCCATTCACTC3’ | 5’CATTCTGTTCTCCACCACCTGCTC3’ |
| *Tgfβ2* | 5’ATTGCTGCCTTCGCCCTCTTTAC3’ | 5’TTGGTGTGTTGTGTGTCTGAACTCC3’ |
| *Lumican* | 5’TCCGCTCCCAAAGTCCCTACAAG3’ | 5’GCCTTTCAGAGAAGCCGAGACAG3’ |
| *Bgn* | 5’GCTTCAGGCTCAGACACCACTTC3’ | 5’GATCTCCTTGGGCACAGTCTTCAG3’ |
| *Serpinh1* | 5’AGCTGCCAGAGGTCACCAAGG3’ | 5’TCATCCCAGTGCGGCTTAAAGAAC3’ |
| *Il-10* | 5’CTGCTATGTTGCCTGCTCTTACTG3’ | 5’GGGTCTGGCTGACTGGGAAG3’ |
| *Col2a1* | 5’GGAGCAGCAAGAGCAAGGAGAAG3’ | 5’TCAGTGGACAGTAGACGGAGGAAAG3’ |
| *Aggrecan* | 5’AATCCAGAACCTTCGCTCCAATGAC3’ | 5’GGTGGCTTCGCTGTCCTCAATG3’ |
| *Mmp13* | 5’ TGCGGTTCACTTTGAGGACA3’ | 5’ GAGGCGGGGATAGTCTTTGT3’ |
| *Mmp3* | 5’ GCGGGGAGAAGTCTTGTTCT3’ | 5’ TGTTGGATGGAAGAGACGGC3’ |
| *Adamts5* | 5’ GGTCAGTGTTCTCGCTCTTG3’ | 5’ GTTAGGTGGGCAGGGTATGA3’ |
| *Cox-2* | 5’ TGACAGCCCACCAACTTACAATG3’ | 5’ TCATCAGCCACAGGAGGAAGG3’ |
| *Tnf-α* | 5’ GCCCAGACCCTCACACTCAG3’ | 5’ CCGCTTGGTGGTTTGCTACG3’ |
| *Anxa1* | 5’ GAAGGGACUUGGAACAGAUTT3’ | 5’ AUCUGUUCCAAGUCCCUUCTT3’ |
